# Deep learning-enhanced automated mitochondrial segmentation in FIB-SEM images using an entropy-weighted ensemble approach

**DOI:** 10.1371/journal.pone.0313000

**Published:** 2024-11-26

**Authors:** Yubraj Gupta, Rainer Heintzmann, Carlos Costa, Rui Jesus, Eduardo Pinho

**Affiliations:** 1 Departamento de Electrónica, Telecomunicações e Informática (DETI), University of Aveiro, Aveiro, Portugal; 2 Leibniz-Institute of Photonic Technology (Leibniz-IPHT), Jena, Germany; 3 BMD Software, Aveiro, Portugal; 4 Institute of Physical Chemistry and Abbe Center of Photonics, Friedrich-Schiller-Universität Jena, Jena, Germany; Soochow University, CHINA

## Abstract

Mitochondria are intracellular organelles that act as powerhouses by breaking down nutrition molecules to produce adenosine triphosphate (ATP) as cellular fuel. They have their own genetic material called mitochondrial DNA. Alterations in mitochondrial DNA can result in primary mitochondrial diseases, including neurodegenerative disorders. Early detection of these abnormalities is crucial in slowing disease progression. With recent advances in data acquisition techniques such as focused ion beam scanning electron microscopy, it has become feasible to capture large intracellular organelle volumes at data rates reaching 4Tb/minute, each containing numerous cells. However, manually segmenting large data volumes (gigapixels) can be time-consuming for pathologists. Therefore, there is an urgent need for automated tools that can efficiently segment mitochondria with minimal user intervention. Our article proposes an ensemble of two automatic segmentation pipelines to predict regions of interest specific to mitochondria. This architecture combines the predicted outputs from both pipelines using an ensemble learning-based entropy-weighted fusion technique. The methodology minimizes the impact of individual predictions and enhances the overall segmentation results. The performance of the segmentation task is evaluated using various metrics, ensuring the reliability of our results. We used four publicly available datasets to evaluate our proposed method’s effectiveness. Our proposed fusion method has achieved a high score in terms of the mean Jaccard index and dice coefficient for all four datasets. For instance, in the UroCell dataset, our proposed fusion method achieved scores of 0.9644 for the mean Jaccard index and 0.9749 for the Dice coefficient. The mean error rate and pixel accuracy were 0.0062 and 0.9938, respectively. Later, we compared it with state-of-the-art methods like 2D and 3D CNN algorithms. Our ensemble approach shows promising segmentation efficiency with minimal intervention and can potentially aid in the early detection and mitigation of mitochondrial diseases.

## Introduction

Cellular organelles play a vital role in the functioning of eukaryotic cells. The mitochondrion has garnered considerable attention among these organelles due to its crucial involvement in various metabolic pathways, including producing Adenosine triphosphate (ATP) [1]. Despite their common depiction as static, bean-shaped structures, mitochondria are highly dynamic, forming tubular networks that continuously change shape and position. They vary in size, ranging from 0.5 to 10 *μm* [2].

Focused ion beam scanning electron microscope (FIB-SEM) imaging is a powerful technique that combines focused ion beam milling with scanning electron microscopy to visualize cellular structures at the nanometer level. It provides detailed 3D geometry of cellular components, including mitochondria, with remarkable precision [3]. FIB-SEM imaging has revolutionized our understanding of cellular architecture by being able to quickly capture significant-high-quality (gigavoxel) intracellular organelle image volumes, offering valuable insights into the size, shape, and organization of organelles like mitochondria [4]. The manual segmentation of mitochondria in FIB-SEM images poses significant challenges. With gigapixel imaging sizes and thousands of slices to process, this manual approach is not feasible and can be highly time-consuming for trained pathologists. Automated segmentation methods are needed to accurately and efficiently extract shape information from these large datasets to overcome this limitation. These advancements in segmentation techniques will significantly enhance our ability to analyze mitochondria and further our understanding of their role in cellular function.

However, automated segmentation and reconstruction of mitochondria in FIB-SEM data comes with its own set of challenges. The diverse range of mitochondrial structures, coupled with the presence of noise, artifacts, and other subcellular structures, adds considerable complexity to the segmentation process [5, 6]. Despite these difficulties, significant progress was made in developing advanced algorithms and techniques to overcome these challenges. Deep learning approaches, feature-based methods, and hybrid strategies that combine multiple strategies have been explored to improve the accuracy and efficiency of mitochondrial segmentation and reconstruction in FIB-SEM data.

### Challenges and limitations in existing methods

Before the advent of deep learning algorithms, traditional machine-learning techniques were used for mitochondria segmentation. For instance, Jorstad et al. [7] proposed an active surface-based method that refined boundary surfaces by leveraging the thick, dark (electron-dense) membranes of mitochondria. The inner membrane served as a reference for accurately delineating the outer membrane surface. Their approach demonstrated higher accuracy in fitting 3D mesh surfaces to mitochondrial membranes than manual annotation. Similarly, Lucchi et al. [8] proposed an approximate subgradient descent algorithm for optimizing margin-sensitive hinge loss in structured support vector machine (SSVM) frameworks for automated image segmentation. Their approach improves convergence, allows random constraint sampling, and achieves state-of-the-art results on electron microscopy datasets.

Likewise, Rigamonti et al. [9] introduced an enhanced kernelBoost classifier that incorporates previous segmentation results and original images iteratively. This recursive approach enables the classifier to focus on challenging regions and effectively leverage the decision tree paradigm, mitigating the risk of overfitting. Cetina et al. [10] conducted a performance comparison of feature descriptors for mitochondria and synapse segmentation, employing Gaussian and Random Forest Classifiers. These methods rely on manually designed features to detect and segment mitochondria. Additionally, Peng et al. [11] introduced an image segmentation method using contextual features called local patch patterns (LPPs) to handle appearance ambiguity in mitochondria datasets. Their approach involves iterative learning of a hierarchical structured contextual forest, incorporating spatial and temporal contexts through a median fusion strategy. They also propose a 2D variant with multi-view fusion for accurate segmentation. However, these methods have limitations regarding generalization and handling large electron microscopy datasets.

### Current approaches to mitochondrial segmentation

Traditional machine learning methods struggle with larger datasets because they rely on manual feature engineering. In contrast, deep learning, mainly using convolutional neural networks (CNNs) [12, 13], has emerged as a powerful approach, inspired by the research of Hubel and Wiesel on the primary visual cortex of cats [14]. CNNs extract features from images using convolution and pooling operations, enabling accurate classification of complex images by applying these operations repeatedly [15].

For semantic segmentation, fully convolutional neural networks (FCNNs) like U-Net [16] are widely used in biomedical image analysis. U-Net architectures were successfully used in various segmentation tasks, even with only a limited amount of hand-segmented images being available [16]. This shift to deep learning has addressed the limitations of traditional methods and improved generalization performance and segmentation accuracy, making it suitable for handling large amounts of electron microscopy data.

Based on existing research, numerous deep-learning techniques have been developed specifically for the automated segmentation of mitochondria. Oztel et al. [17] introduced a fully convolutional network (FCN) with four convolutional layers, three pooling layers, and one fully connected layer for automated mitochondria segmentation. They further improved the segmentation results by applying post-processing techniques, including 2D spurious detection filtering, boundary refinement, and 3D filtering. Similarly, the SyConn framework was introduced by Dorkenwald et al. [18], employing recursive deep convolutional neural networks and random forest classifiers to locate mitochondria. The primary objective of their framework is to infer a comprehensive synaptic connectivity matrix by automatically identifying different cellular components based on manual neurite skeleton reconstructions. However, it is worth noting that the accuracy of mitochondria segmentation within the framework was not deemed satisfactory, as the main focus was on synapse segmentation.

Furthermore, Liu et al. [19] introduced a technique that employs Mask region-based (R-CNN) [20] for segmenting FIB-SEM images. Their significant contribution focuses on the post-processing phase of the obtained segmentation masks using the deep network. The post-processing steps involve eliminating small regions, smoothing large regions through morphological opening, removing shorter mitochondria using a 3D multi-layer fusion algorithm, and enhancing consistency across adjacent layers. Similarly, Casser et al. [21] introduced a modified version of the 2D U-Net [16] designed explicitly for real-time segmentation. Their approach includes reducing the number of convolutional filters, replacing transpose convolutions with lightweight bilinear upsampling layers, and using padding layers instead of center-cropping to maintain dense output at full resolution. Additionally, in the post-processing stage, they applied the median Z-filtering method.

The approaches mentioned above, including Oztel et al. [17], Dorkenwald et al. [18], Liu et al. [19], and Casser et al. [21] did not incorporate 3D spatial information in their network training process, relying exclusively on 2D convolutions.

However, some publications specifically concentrate on developing 3D automated segmentation pipelines for mitochondria. For instance, Xiao et al. [2] proposed a method utilizing a modified version of the 3D U-Net [22] model with residual blocks. They addressed the vanishing gradient problem by incorporating two auxiliary outputs in the decoder section. Their ensemble prediction approach involved considering 16 possible 3D variations, achieved through flips and axis rotations, for each 3D subvolume. To address previous studies’ limitations in utilizing labeled and unlabeled data for mitochondria segmentation, Yuan et al. [23] proposed a multi-task network named EM-Net. Their approach includes an auxiliary centerline detection task to capture the shape information of mitochondria, even with limited labeled data, resulting in improved accuracy and robustness in segmentation. However, it is worth noting that these studies did not effectively leverage large volumes of unlabeled data for model training.

Yuan et al. [24] proposed HIVE-Net, an interconnected multi-branch architecture that decomposed 3D convolutions into 2D convolutions. This approach achieved state-of-the-art performance while maintaining a compact model size and low computational requirements. Mekuč et al. [5] proposed the HighRes3DZMNet pipeline, a modified version of the HighRes3DNet [25] architecture, for automated segmentation of mitochondria and endolysosome regions. As part of their approach, they addressed the issue of varying brightness within the image by modifying the input layer of the algorithm, incorporating a zero-mean convolution layer.

In a recent study, Guo et al. [26] developed a two-stage cascaded architecture that combines top-down and bottom-up approaches for mitochondria segmentation. The first stage focuses on precise contour delineation through object localization, while the second stage considers 3D connectivity for accurate segmentation. Segmentation cues generated from multi-slice fusion in the detection stage are used to supervise the 3D CNN segmentation in the second stage, incorporating pixel properties and 3D connectivity information. Pan et al. [27] present an adaptive template transformer (AT-Former) combined with a hierarchical attention learning mechanism for mitochondria segmentation. The templates are used to accurately learn specific semantic information about the data, such as the images’ background, foreground, and contour. The hierarchical attention mechanism allows the algorithm to work at different scale levels of the image, granting the algorithm better performance results at different scales.

Similarly, Luo et al. [28] proposed a coherent fragment vision transformer (FragViT) that utilizes affinity learning to manipulate features on 3D fragments while exploring mutual relationships to model fragment-wise context. FragViT includes a fragment encoder and a hierarchical fragment aggregation module. The fragment encoder transforms the tokens into fragments with homogeneous semantics using affinity heads, and multi-layer self-attention is used to learn inter-fragment relations with long-range dependencies. The hierarchical fragment aggregation module aggregates fragment-wise predictions back to the final voxel-wise prediction in a progressive manner. This approach allows the model to maintain locality prior without sacrificing global reception.

Advancements in 2D and 3D deep learning algorithms for segmentation techniques are promising in enhancing our understanding of mitochondria and their functions. The integration of such automated segmentation methods with FIB-SEM imaging data holds the potential to unlock valuable insights into mitochondrial dynamics, structure, and interactions. This has significant implications for fields like neuroscience and clinical studies, offering a better understanding of conditions such as bipolar disorder and diabetes.

### Addressing gaps with ensemble approaches

In this article, we propose a simple and effective post-processing technique that combines the predicted outputs from Mask R-CNN [20] and 3D U-Net [22] networks using an ensemble learning-based entropy-weighted average linear fusion method. This approach allows us to generate more accurate segmentation results by taking advantage of both object detection and pixel-wise segmentation networks.

Our motivation for adopting these methods is based on their demonstrated advantages in other biomedical research, where they have successfully segmented a high percentage of foreground regions of interest compared to other deep models. To ensure that Mask R-CNN and 3D U-Net were the most suitable choices for our ensemble method, we evaluated their performance alongside four other advanced networks, Ddrnet-23 [29], Segformer-B2 [30], YoloV8 [31], and YoloV9 [32], on four publicly available mitochondrial datasets: (1) Lucchi, (2) Lucchi++, (3) Kasthuri++, and (4) UroCell.

Our comparative analysis showed that Mask R-CNN and 3D U-Net consistently outperformed the other models on these datasets, thereby justifying their selection for our ensemble approach. Another key focus of our study was to develop a pipeline capable of accurately segmenting the different structures of mitochondria. We evaluated our proposed method on the aforementioned mitochondrial datasets with varying mitochondrial structures to achieve this. We then compared our results to other promising studies that have conducted similar work on these datasets.

In summary, this work makes two main contributions:

- *Method*: We have designed an automated pipeline for segmenting mitochondria in isotropic FIB-SEM images. Our method combines two computer vision techniques: object detection with segmentation using Mask R-CNN [20] and semantic image segmentation using the 3D U-Net [22] architecture. To account for the diverse shapes of mitochondria, we have introduced a post-processing stage where we merge the predicted outputs of both models using an ensemble learning weight fusion strategy. While Mask R-CNN and 3D U-Net are well-established models, their unique combination tailored for this specific task represents a significant contribution. The innovative aspect of our approach is the entropy-weighted fusion strategy, which dynamically adjusts weights based on model performance metrics, enhancing segmentation accuracy and robustness. Our approach significantly advances the current state-of-the-art by providing a more accurate and reliable method for mitochondrial segmentation, as demonstrated by our superior performance metrics compared to existing methods. This enables us to reconstruct complete mitochondrial shapes. Finally, we compare the performance of our proposed fusion strategy with previously published state-of-the-art results. Overall, our approach aims to improve the accuracy and completeness of mitochondrial segmentation by leveraging the strengths of both Mask R-CNN [20] and 3D U-Net [22] through our fusion post-processing strategy.
- *Varying mitochondria structures*: To assess the effectiveness of our proposed method in segmenting varying mitochondrial structures, we conducted experiments on four publicly available datasets: Lucchi, Lucchi++, Kasthuri++, and UroCell. The selection of these datasets was deliberate, as they encompass a wide range of mitochondrial structures and imaging conditions. Moreover, their extensive use in existing research provides a solid benchmark to evaluate our model’s performance. This comprehensive approach allowed us to evaluate our method’s robustness and generalization capabilities across diverse scenarios.

The structure of our article is designed to provide a comprehensive understanding of our research. The Materials and Methods section describes the datasets used and outlines our unique approach. Next, we present the experimental results and discuss the findings comprehensively, providing a thorough analysis of our method’s performance. Finally, the conclusion summarizes the key points and offers insights into future works, ensuring the reader is fully informed about our research and its implications.

## Materials and methods

In this section, we will provide an overview of the datasets used in our experiments, followed by a summary of how the Mask R-CNN [20] and the 3D U-Net [22] models function. Subsequently, we will delve into a detailed description of our proposed pipeline.

### Image volume datasets

Our experiment utilized four publicly available mitochondrial datasets, each containing a diverse range of mitochondrial structures, including oval and tube-like shapes. We had the input and corresponding labeled images for each dataset, which domain experts annotated. We selected these datasets for their high quality and the extensive research conducted using them, which provides a benchmark for comparing our work.

**Fig 1:**
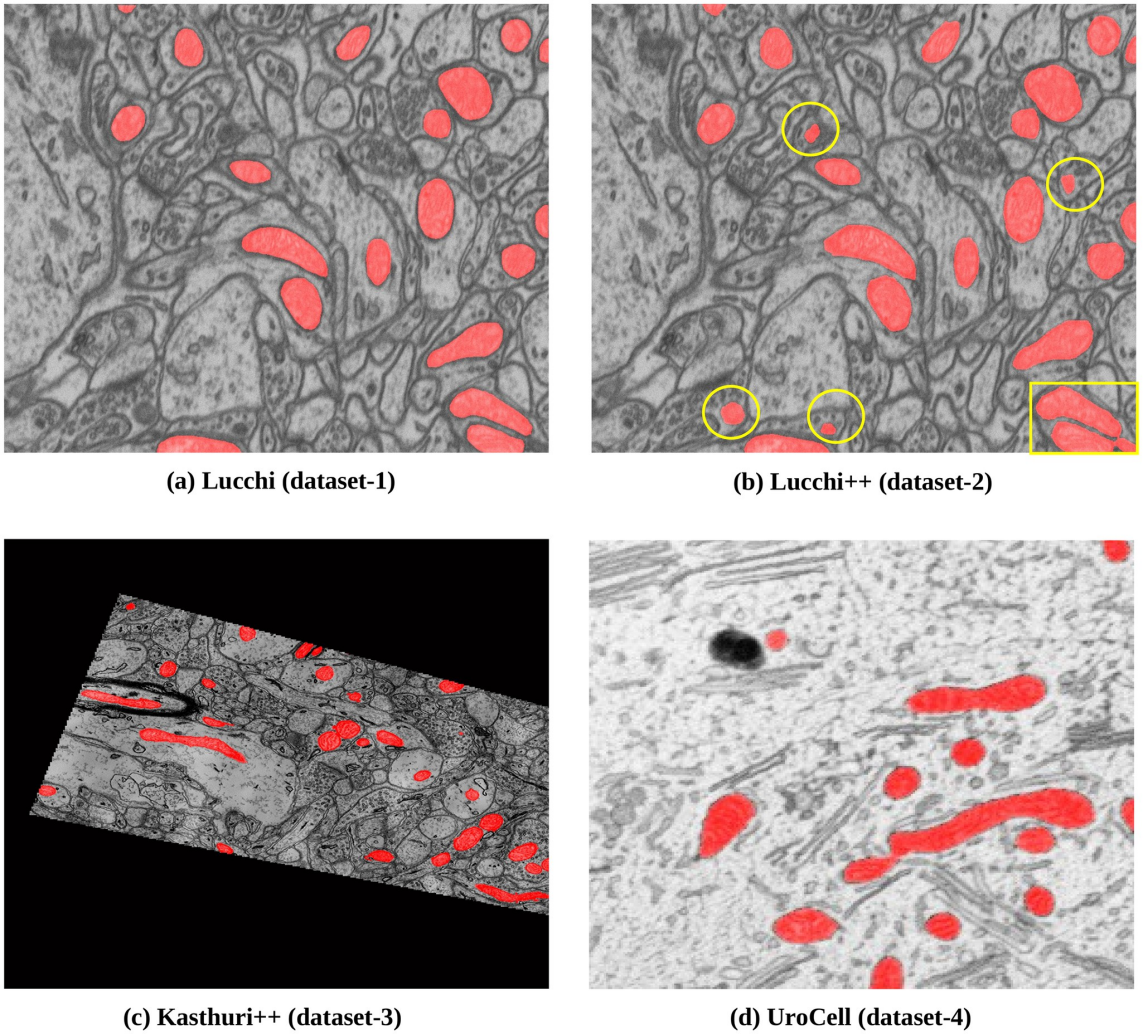
Four publicly available datasets, (a) Lucchi, (b) Lucchi++, (c) Kasthuri++, and (d) UroCell were used in this study. Each dataset was overlaid with its corresponding (ground-truth) label data, annotated by domain experts. For dataset-1(a) and dataset-2(b), different experts annotated the same slice, and the differences in annotations were highlighted using a yellow circle.

- *Lucchi (dataset-1)*: The dataset used in this study was obtained from the EPFL homepage (https://www.epfl.ch/labs/cvlab/data/data-em/) [33]. The original image volume represents a section of the CA1 hippocampus region of a mouse brain, measuring 5 x 5 x 5 *μm* with a voxel resolution of 5 x 5 x 5 nm. The FIB-SEM microscope was used to acquire a total of 2048 x 1536 x 1065 voxels. On their homepage, the dataset consists of two subvolumes for training and testing, containing 165 slices of images with dimensions of 1024 x 768 pixels each. These images are accompanied by corresponding labeled data, which have been expertly annotated. Fig 1a illustrates the Lucchi dataset, where the image is overlapped with its corresponding labeled image.
- *Lucchi++ (dataset-2)*: This dataset was obtained from the Connectomics homepage (https://sites.google.com/view/connectomics). Dataset-2, also known as Lucchi++ [21], is an updated version of Dataset-1 (Lucchi) [33]. The decision to create Lucchi++ was made by [21] and a neuroscientist due to observed boundary inconsistencies in the ground-truth annotations provided in Dataset-1. To address this issue, two experienced senior neuroscientists re-annotated the data. Fig 1b visually demonstrates the difference in annotation between Lucchi++ [21] and Lucchi (Fig 1a) [33] for the same slice, highlighted by a yellow circle.
- *Kasthuri++ (dataset-3)*: The Kasthuri++ dataset (dataset-3) was also downloaded from the Connectomics homepage (https://sites.google.com/view/connectomics). This dataset is a re-annotation of the dataset provided by [34]. The volumes in [34] correspond to the S1 primary somatosensory cortex of the 3-cylinder mouse cortex volume, acquired using a serial section electron microscopy (ssEM) device. Like the Lucchi++ dataset, [21] enlisted the help of the same two experienced senior neuroscientists to re-annotate these two volumes. The training volume has dimensions of 1463 x 1613 x 85 voxels, and the testing volume has dimensions of 1334 x 1553 x 75 voxels, with a voxel size of 3 x 3 x 3 nm. Fig 1c illustrates the Kasthuri++ dataset, where the image is overlapped with its corresponding labeled image.
- *UroCell (dataset-4)*: The UroCell dataset was downloaded from the webpage provided by [5] (https://github.com/MancaZerovnikMekuc/UroCell). This dataset consists of volumes obtained from the urinary bladder of male mice using a FIB-SEM dual-beam electron microscope device. The authors have provided eight sub-volumes, out of which five sub-volumes include both the input images and their respective annotated data. The remaining three sub-volumes (fib1-2-2-3, fib1-2-3-0, and fib1-2-3-2) contain raw images without annotations. Each sub-volume has dimensions of 256 x 256 x 256 voxels, with a voxel size of 16 x 16 x 15 nm. Fig 1d in the paper illustrates the UroCell dataset, showing the image overlapped with its corresponding labeled image.

### Mask R-CNN & 3D U-Net

**Fig 2:**
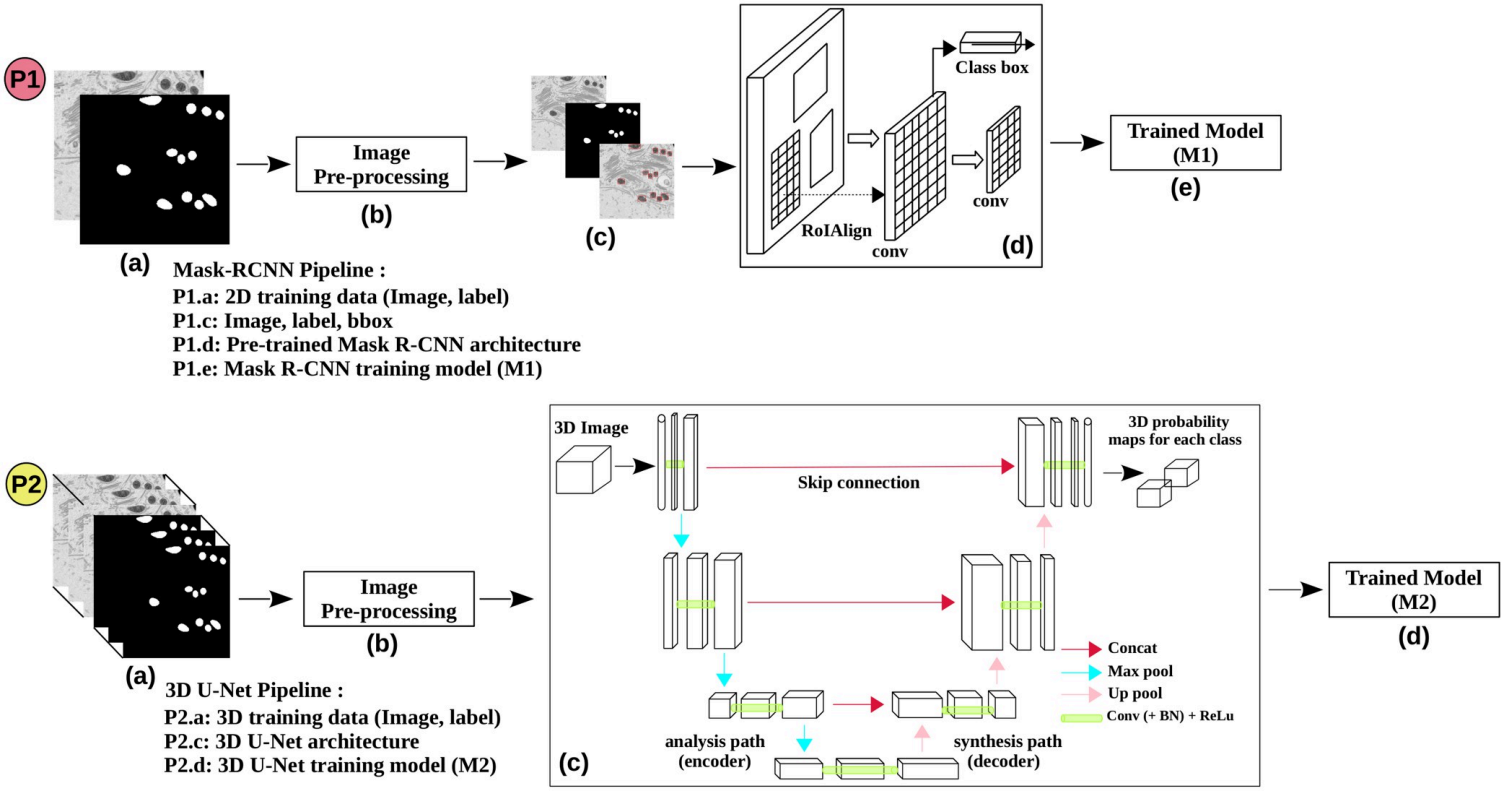
Proposed pipeline for the training and testing using the four datasets. (P1) represents the working pipeline for Mask R-CNN, while (P2) illustrates the image segmentation pipeline for the 3D U-Net architecture. The pipeline outlines the sequential steps involved in preprocessing, training, and saving a trained model for each dataset.

The Mask Region-based CNN (R-CNN) [20] is a state-of-the-art convolutional neural network (CNN) model used for image segmentation tasks. It is a variant of the Faster R-CNN [35] architecture, a popular computer-vision object detection algorithm. The Mask R-CNN [20] detects objects in an image and generates precise pixel-to-pixel segmentation masks for each instance. While the majority of layers in Mask R-CNN [20] is the same as in Faster R-CNN [35], it incorporates three additional layers: MaskHeads, MaskPredictor, and MaskRoiPool as shown in Fig 2 (P1) d. These extra layers facilitate the segmentation of detected objects. In Faster R-CNN [35], the user provides the input image and labeled bounding boxes and expects to receive two outputs: the bounding boxes for each object and their corresponding class labels with confidence scores. However, in Mask R-CNN [20], the user must provide the input image, labeled bounding boxes, and corresponding mask images. As a result, the user receives three outputs: the bounding boxes for each object, class labels with confidence scores for each detected box, and the segmentation masks. Mask R-CNN architectures, as introduced by [20], have been widely adopted in biomedical research studies [19, 26, 36], and similarly, we leverage these architectures in our work.

The 3D U-Net [22] architecture is an extension of the 2D U-Net [16] architecture, specifically designed to handle three-dimensional volumetric inputs (h x w x d) for pixel-wise image segmentation tasks as shown in Fig 2(P2) c. Similar to the 2D U-Net [16], the 3D U-Net [22] consists of both a 3D contractive (encoder) and a 3D expanding (decoder) structure. The 3D contractive structure takes the 3D volume as input and performs dimensionality reduction using 3D max-pooling layers. This structure aims to create a bottleneck in the center part of the network, where the spatial information is condensed through a combination of 3D convolution and pooling operations. On the other hand, the 3D expanding structure performs upsampling operations to reconstruct the segmented image. It achieves this by combining 3D convolutions and 3D upsampling layers to restore the original resolution of the input volume. Similar to the 2D U-Net [16], the 3D U-Net [22] architecture has been widely used in various biomedical research studies [2, 26, 37] for tasks such as image segmentation and as well as for boundary prediction, leveraging its ability to capture spatial information in three dimensions.

### Proposed method

The proposed method workflow consists of three main subsections: pre-processing, training and testing, and post-processing. These subsections are integral parts of the overall working pipeline of the method, as illustrated in Fig 2, where two deep neural networks are depicted: (P1) represents the pipeline for the Mask R-CNN, and (P2) represents the pipeline for the 3D U-Net. The pre-processing stage involves preparing the input data, while the training and testing process focuses on training the models and evaluating their performance of testing data. Finally, the post-processing stage is applied to refine the segmentation results obtained from the earlier workflow. The clear visual representation in Fig 2 helps to illustrate the different stages and the involvement of the two deep neural networks in the proposed method workflow.

#### Pre-processing

**Fig 3:**
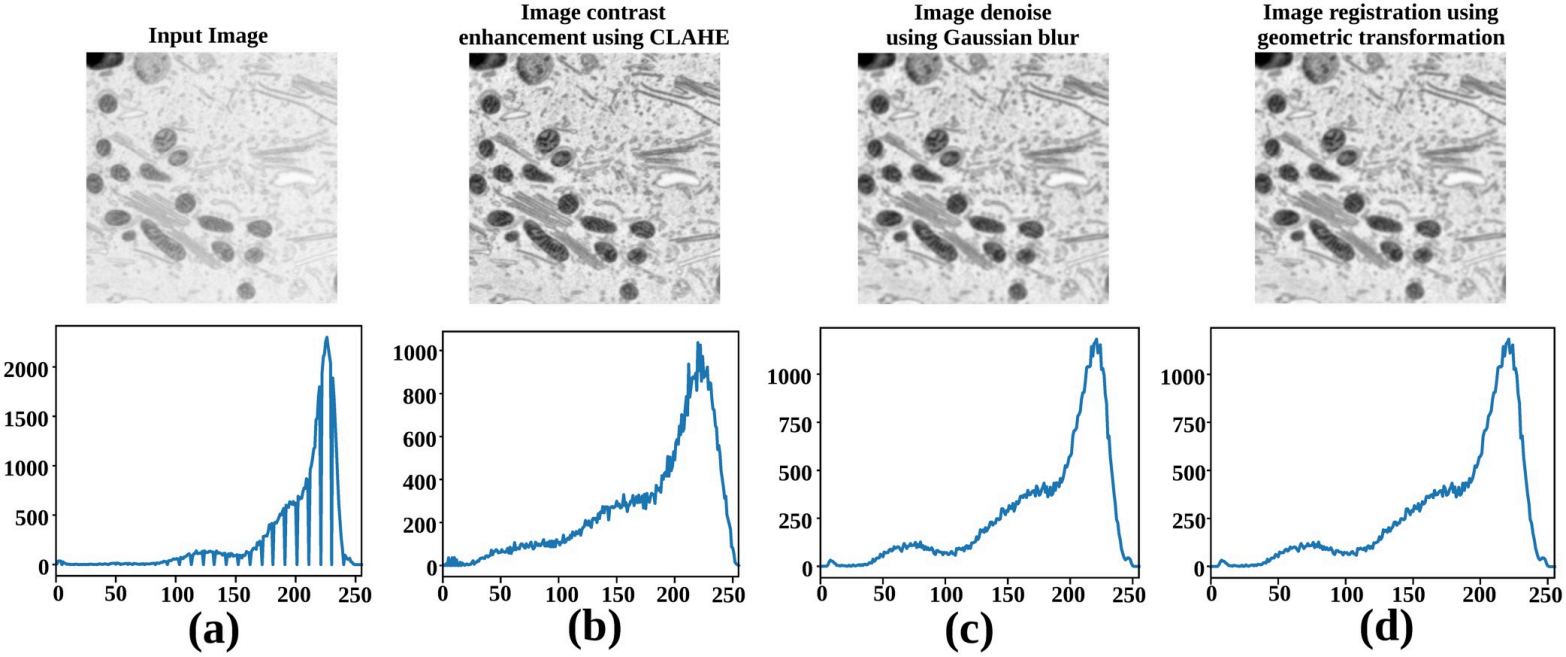
Pre-processing steps. Dataset-4 (UroCell) undergoes several pre-processing stages before being fed into six different selected deep neural networks.

In the pre-processing phase, the 3D volume input data undergoes multiple steps before feeding into the training model (Fig 2), as illustrated in Fig 3. First, contrast enhancement employs the contrast-limited adaptive histogram equalization (CLAHE) method. Our implementation uses a clip limit of 2 and an 8 x 8 tile grid size, balancing contrast enhancement while preserving image details (Fig 3b). Next, Gaussian blur with a small 3 x 3 kernel is applied to reduce noise, maintaining image integrity (Fig 3c). Lastly, a geometric transformation aligns volume slices for accurate segmentation (Fig 3d). These steps prepare the pre-processed input volumes for training and testing with the Mask R-CNN and 3D U-Net neural network models.

#### Training and testing process

The training and testing process involves initializing, training, and optimizing deep learning models on pre-processed data. This iterative process improves model performance through parameter adjustments via backpropagation. Once trained, the models are assessed on new, unseen data to gauge segmentation accuracy and generalizability.

Our approach includes training separate models for the four datasets using Mask R-CNN and 3D U-Net networks. This individualized training per dataset aims to capture distinct mitochondrial data characteristics, enhancing segmentation performance specific to each dataset.

In our Mask R-CNN pipeline (Fig 2P1), we constructed custom dataset file readers tailored to each dataset. These readers facilitated image and mask reading, object counting, and bounding box generation on the original image without resizing them for training, thereby preserving the original image dimensions. Our model utilized the “maskrcnn_resnet50_fpn” architecture, integrating a Feature Pyramid Network (FPN) for multi-scale feature extraction. We employed the Stochastic Gradient Descent (SGD) optimizer to optimize model parameters with a learning rate of 0.005, momentum of 0.9, and weight decay of 0.005. Additionally, we utilized a stepLR scheduler, reducing the learning rate by 0.1 every three epochs. The choice of 100 epochs for training was based on a balance between achieving effective learning and computational efficiency.

**Fig 4:**
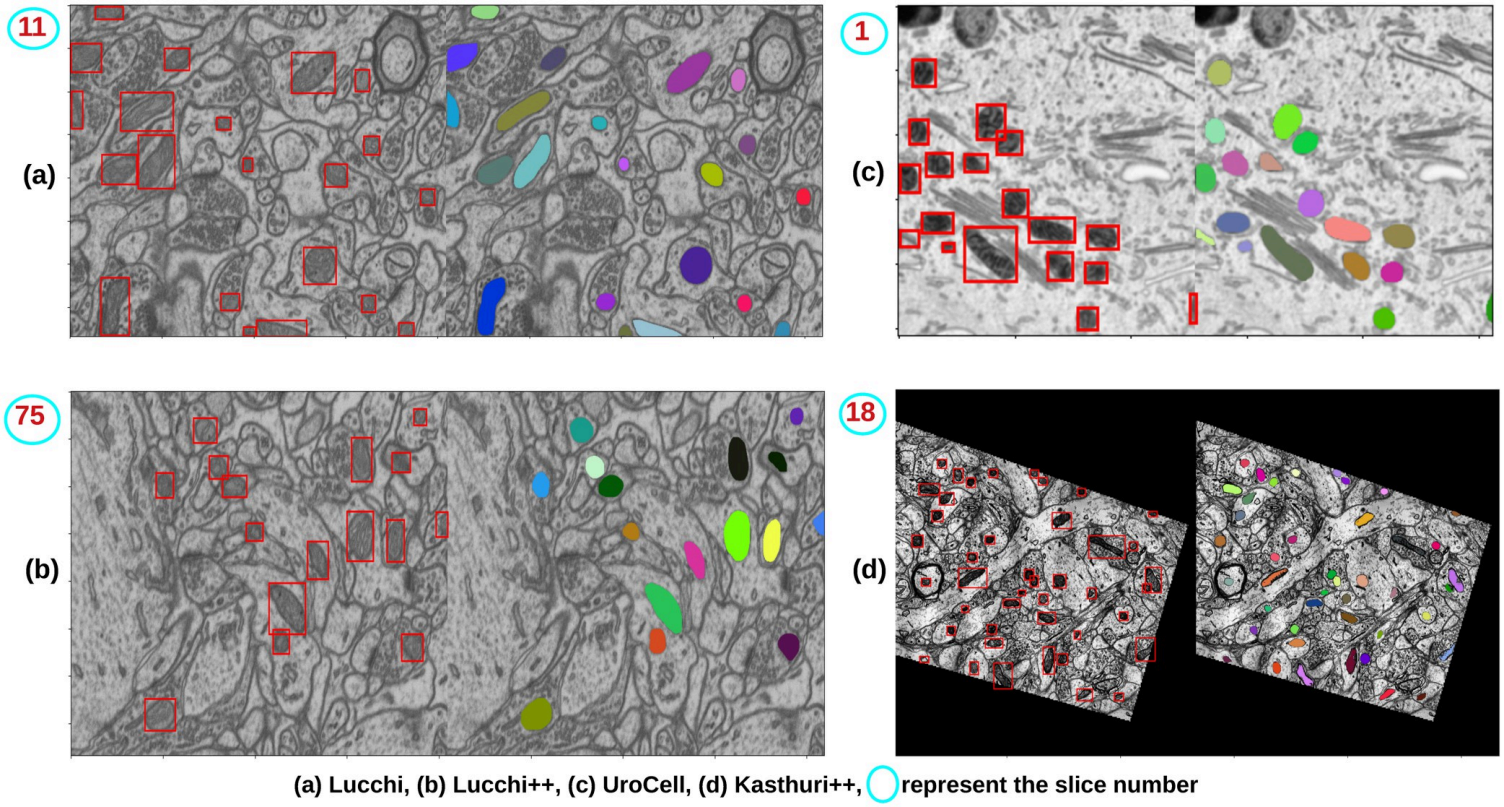
The image shows the predicted boundary boxes (in red) generated along with their respective segmented images (in random color) after passing each volume slice through the Mask R-CNN trained model (M1).

The model utilized data augmentation during training to prevent overfitting, and it was exclusively trained on the provided dataset. A separate validation set, comprising 20% of the data, enabled ongoing performance evaluation on unseen data ([5, 21, 33, 34]). After completing 100 epochs, models were saved for subsequent test image predictions. Implementing this inference process for each dataset, we obtained predicted binary masks for all data (refer to Fig 4).

After the Mask R-CNN pipeline, we applied the pre-processed data to the 3D U-Net pipeline (Fig 2P2) using Pytorch-3dunet (https://github.com/wolny/pytorch-3dunet) based on [38]. Instead of building our own 3D U-Net pipeline from scratch, we used the pytorch-3dunet library due to its proven performance in various research papers. In their library, [38] implemented three semantic segmentation algorithms, including 3D U-Net [22], ResidualUNet3D [39], and ResidualUNetSE3D [40]. We attempted to use both 3D U-Net and ResidualUNet3D from this library for our experiment. However, we found that ResidualUNet3D did not yield satisfactory results. As a result, we proceeded to run each dataset through the 3D U-Net network only.

The train-and-test configuration YAML file defined the model, loss function, optimizer, and learning rate scheduler. We utilized BCEDiceLoss as the loss function, combining binary cross-entropy and dice losses. The Adam optimizer with a learning rate of 0.0002 and weight decay of 0.00001 was chosen. Employing the ReduceLROnPlateau scheduler, we monitored metric improvements and reduced the learning rate by 0.2 if no improvement was observed within ten epochs, optimizing resources. Training for 1000 epochs used a patch shape of 64 x 64 x 64 for each data point, accompanied by data augmentation techniques (flipping, rotation, elastic deformation) to prevent overfitting. The 3D U-Net architecture processes volumetric inputs, reduces dimensionality through convolutions and pooling, and reconstructs segmented images using convolutions and upsampling layers.

**Fig 5:**
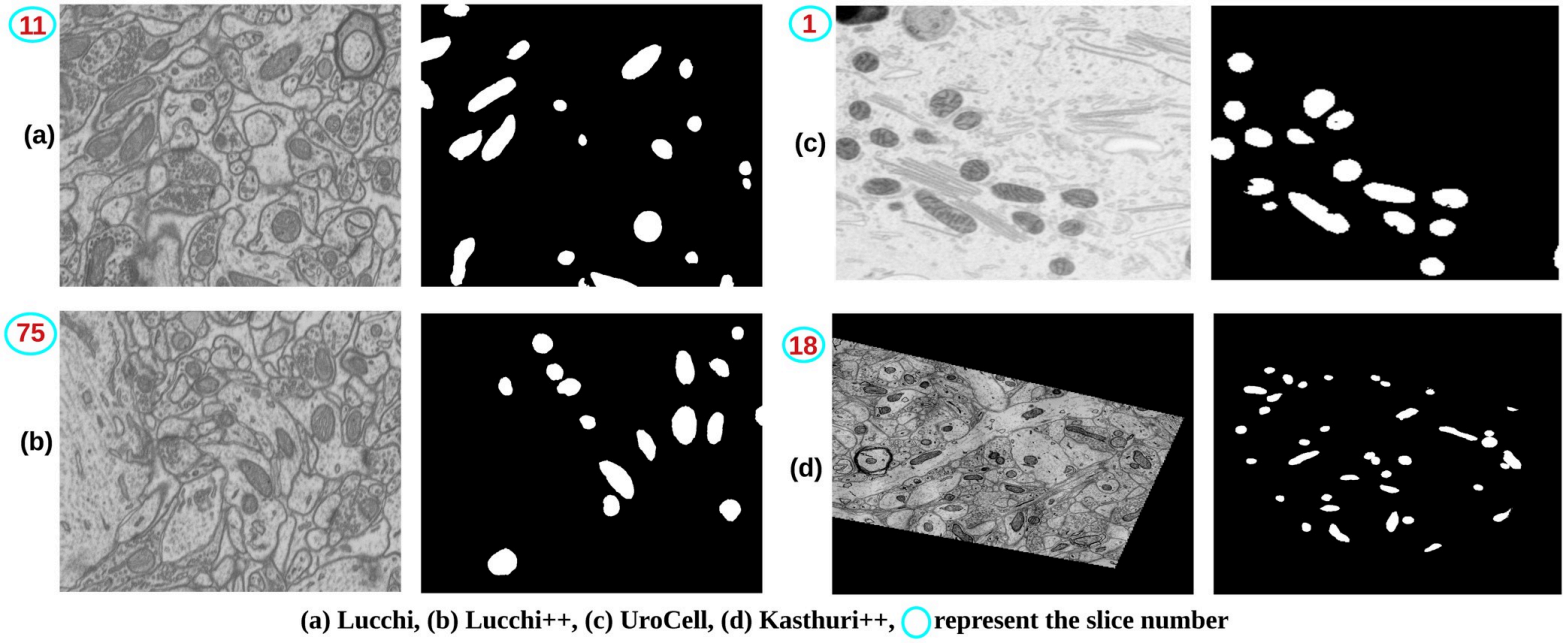
The image shows the predicted mask generated for all four datasets after passing each volume through a 3D U-Net trained model (M2).

After completing the training phase, we saved each dataset’s trained 3D U-Net model (M2). We employ the saved models (M2) by passing the test image volume through the inference pipeline to make predictions on unseen test image volumes. This process involves inputting the test volume into the trained model and generating a predicted binary mask. Repeating this inference for each dataset results in predicted binary masks, as illustrated in Fig 5.

#### Post-processing

**Fig 6:**
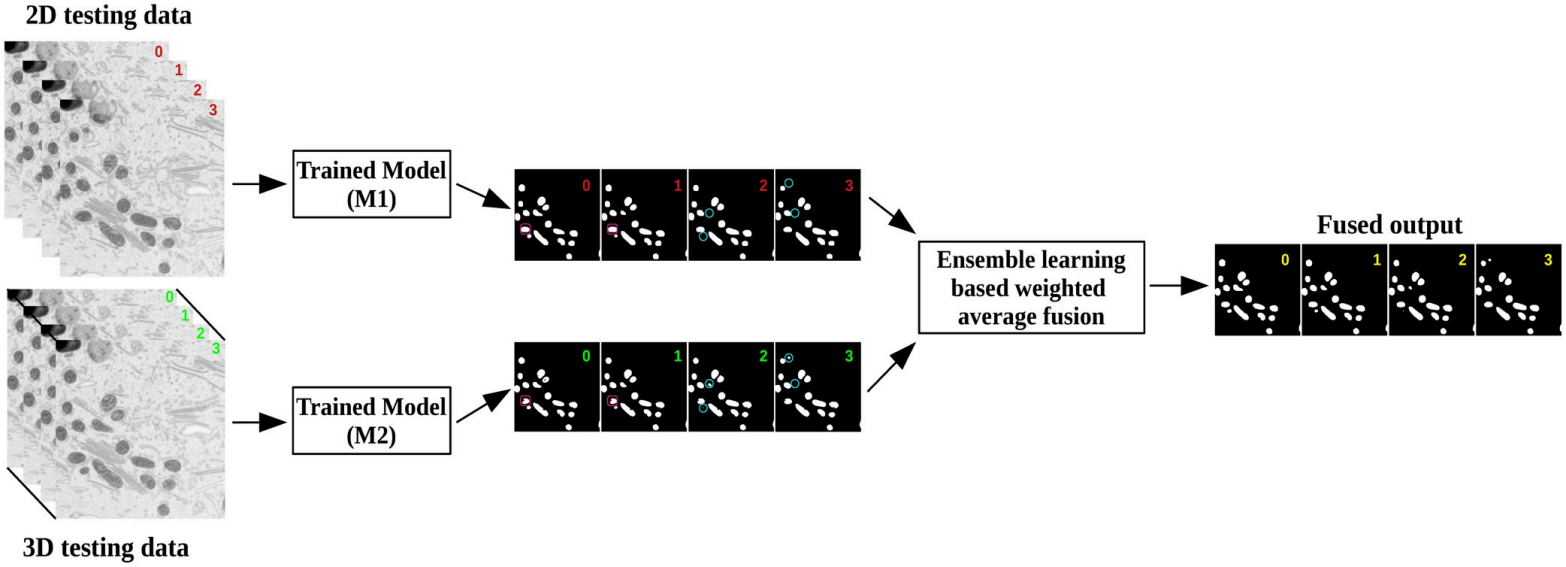
Post-processing stage. The image illustrates the final segmentation result obtained by combining the predicted outputs from each trained model (M1) and (M2), Mask R-CNN, and 3D U-Net models, using the ensemble learning weight average fusion technique. This fusion process enhances the accuracy of the segmentation and produces the final prediction output.

In our post-processing phase, we aim to refine the segmentation results obtained from modes P1 and P2. Instead of traditional techniques like thresholding or morphology operations, we adopt a fusion strategy based on ensemble learning [41], illustrated in Fig 6. This weighted average fusion method aims to utilize the best features of each model to create a more detailed and accurate segmentation.

Ensemble learning usually assigns weights to models based on data characteristics. We use an entropy weight measure method, a scientific approach that considers data specifics to determine weights. This method assesses variables or indices based on entropy values, reflecting their disorder or information content. Higher entropy variables receive smaller weights, while lower entropy variables get larger weights. This approach objectively assigns weights, considering discrimination ability and offering a deeper understanding of data integration dynamics. This fusion technique linearly combines the model outputs based on their entropy-determined weights, providing a strategic advantage over simple average voting methods.

In average voting, each model in the ensemble contributes equally to the final prediction, regardless of its performance or reliability. This can be problematic when some models are more accurate or relevant to specific data segments than others, as average voting does not differentiate between the quality of input models. It treats each model’s prediction with the same level of importance, potentially diluting the influence of more accurate models with those of lesser accuracy.

Conversely, the linear model approach adjusts the influence of each model’s prediction according to its weight, determined by entropy measures.

**Fig 7:**
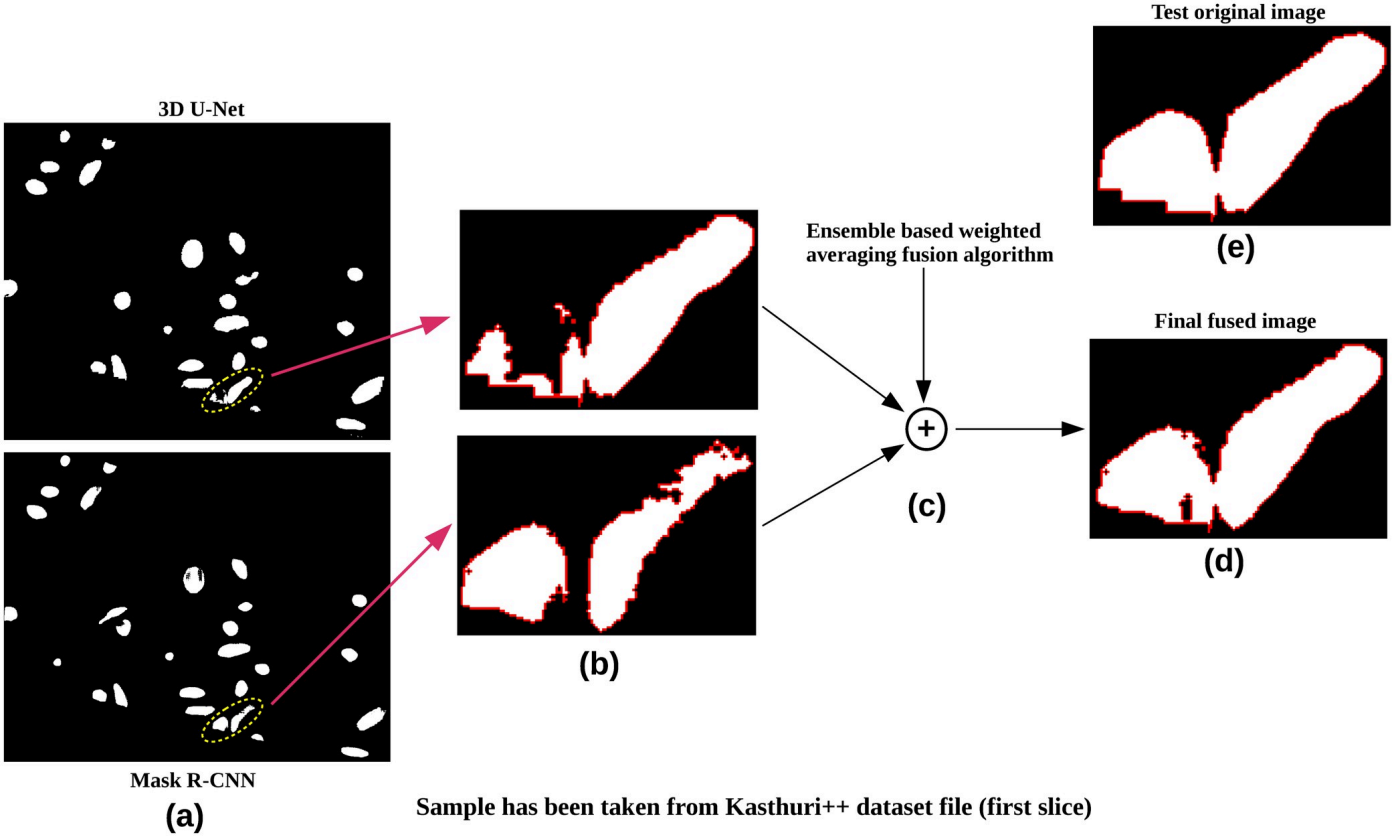
The image represents the results of passing the first slice of the Kasthuri++ testing data through trained M1 and M2 models to obtain their respective predicted outputs. In (a), the predicted outputs from both models exhibit false negatives, indicated by the yellow circles within the image. (b) shows the one found false negative object. To address this limitation, we propose an ensemble learning weight averaging fusion method, shown in (c), which combines the outputs based on the weights obtained using entropy. The resulting new segmentation result, displayed in (d), demonstrates the effectiveness of this technique in overcoming the limitations of individual models. Finally, the segmentation result in (d) is visually compared with the expert-annotated label image (e).

Employing entropy-weighted linear fusion directly addresses the limitations of individual models. Specifically, it compensates for areas where a model may produce false negatives, effectively filling in gaps in the segmentation. This tailored combination not only leverages the unique strengths of each model but also mitigates its weaknesses, leading to a more accurate and complete segmentation, as shown in Fig 7. This strategy significantly boosts the segmentation’s accuracy and completeness, offering a deeper and more holistic understanding of the analyzed objects. Importantly, this method dynamically adjusts weights to enhance overall segmentation accuracy, reassuring us about the adaptability of our approach.

The proposed ensemble learning-based entropy-weighted average fusion can be defined using the below expressions:

In our case, we have two models, let us assume;

$$\begin{array}{c} m_{n}=\text{prediction}\;\text{output}\;\text{of}\;model_{n} \end{array}$$ (1)

Where *n* is the number of individual models (*n* = 2 in this study, as we have utilized two models in this experiment),

$$m_{mask\_rcnn}=\text{prediction}\;\text{output}\;\text{of}\;model_{mask\_rcnn},$$

$$m_{3d-unet}=\text{prediction}\;\text{output}\;\text{of}\;model_{3d-unet},$$

And evaluation metrics for each model;

$$\begin{array}{c} Em_{l}=\text{model}\_\text{metrics}\;\text{of}[“model_{l}”] \end{array}$$ (2)

Where *l* is the number of individual model evaluation metrics, such as the score of *Jaacard*_*Foreground*_, *mean*_*IoU*, *precision*, *recall*, *dice*, etc.

Utilizing (2), we can have *Em*_*mask*_*rcnn*_ and *Em*_3*d*−*unet*_

$$Em_{mask\_rcnn}=\text{model}\_\text{metrics}\;\text{of}\;[“model_{mask\_rcnn}”]$$

$$Em_{3d-unet}=\text{model}\_\text{metrics}\;\text{of}\;[“model_{3d–unet}”]$$

First, we need to calculate the entropy for each model, which can be calculated by using the obtained evaluation metrics of each model;

$$\begin{array}{c} Entropy_{model}=-\Sigma \left(p\;log_{2}\left(p\right)\right) \end{array}$$ (3)

Where *p* represents the probability of each metric for the model.

By utilizing Eq (3), we can calculate the entropy for each model as follows;

$$Ent_{mask\_rcnn}=Entropy_{model}\left(Em_{mask\_rcnn}\right)$$

$$Ent_{3d-unet}=Entropy_{model}\left(Em_{3d-unet}\right)$$

Now, after calculating the entropy, we can calculate the weight for each model by the below expression;

$$\begin{array}{c} Weight_{model}=\frac{1-Entropy_{model}}{\Sigma (1-Entropy_{model})} \end{array}$$ (4)

Now, utilizing (4), we can calculate *Wt*_1_ and *Wt*_2_ for Mask-R-CNN and 3D U-Net;

$$Wt_{mask\_rcnn}=\frac{1-Ent_{mask\_rcnn}}{\left(1-Ent_{mask\_rcnn}\right)+\left(1-Ent_{3d-\;unet}\right)}$$

$$Wt_{3d-unet}=\frac{1-Ent_{3d-unet}}{\left(1-Ent_{mask\_rcnn}\right)+\left(1-Ent_{3d-\;unet}\right)}$$

In the next step, we have to normalize the weight;

$$Wt_{mask\_rcnn,\;normalized}=\frac{Wt_{mask\_rcnn}}{Wt_{mask\_rcnn}+Wt_{3d-unet}}$$

$$Wt_{3du-net,\;normalized}=\frac{Wt_{3d-unet}}{Wt_{mask\_rcnn}+Wt_{3d-unet}}$$

Now, we can pass these normalized weights for each respective model to perform ensemble learning weighted averaging fusion;

$$\begin{array}{c} \begin{array}{c} P=(Wt_{mask\_rcnn,\;normalized}\times m_{mask\_rcnn})\\ +(Wt_{3d-unet,\;normalized}\times m_{3d-unet}) \end{array} \end{array}$$ (5)

The expression (5) signifies the ensemble learning weighted average fusion using entropy weights, denoted by *P* as the ensemble prediction and (×) representing multiplication. This fusion method linearly combines the predictions of two models based on their respective weights.

### Evaluation metrics

In our evaluation phase, we rigorously computed several metric scores to assess the performance of our segmentation models thoroughly. After processing the test data through individually trained models, M1 and M2, and subsequently employing our proposed ensemble learning method, we meticulously calculated a range of metrics. These metrics were carefully chosen to gauge the segmentation results comprehensively and provide a nuanced understanding of the models’ effectiveness in identifying regions of interest.

- **Area Under the Receiver Operating Characteristic (AUROC):** The AUROC value quantifies the overall ability of a model to distinguish between the two classes (usually positive and negative) across different classification thresholds. A higher AUROC score indicates better model performance regarding classification accuracy and the ability to discriminate between the classes. The AUROC curve represents the trade-off between a true positive rate (sensitivity) and a false positive rate (1-specificity) at various threshold values.
- **Pixel Accuracy:** It is the ratio of correctly classified pixels to the total number of pixels in the segmentation mask. Pixel Accuracy is a simple and intuitive evaluation metric used in image segmentation tasks to assess the accuracy of pixel-level predictions made by a segmentation model. It measures the percentage of correctly classified pixels in the predicted segmentation mask compared to the corresponding pixels in the ground truth mask. This metric provides a straightforward way to gauge the overall accuracy of a segmentation model’s predictions. Pixel Accuracy values range from 0 to 1, where 1 indicates perfect pixel-level accuracy (all pixels are correctly classified), and 0 indicates no pixel-level accuracy (none of the pixels are correctly classified).
  $$\begin{array}{c} \text{Pixel}\;\text{Accuracy}=\frac{\text{Correctly}\;\text{classified}\;\text{pixels}}{\text{Total}\;\text{pixels}} \end{array}$$ (6)
- **Error Rate:** It is a metric that quantifies the percentage of incorrectly classified samples in a dataset and complements accuracy. To calculate it, we need to know the number of misclassified samples (i.e., the number of samples classified incorrectly), which refers to the count of samples classified incorrectly by a model or system. These are instances where the predicted class or label does not match the true class or label and the total number of samples in the dataset (this represents the entire set of samples or instances you are evaluating. It includes correctly and incorrectly classified samples).
  $$\begin{array}{c} \text{Error}\;\text{Rate}=\frac{\text{Number}\;\text{of}\;\text{misclassified}\;\text{samples}}{\text{Total}\;\text{number}\;\text{of}\;\text{samples}} \end{array}$$ (7)
  In summary, the error rate measures how well a model or system performs in classifying samples correctly. A lower error rate indicates better performance, while a higher error rate suggests that more samples are misclassified.

Besides the above metrics, we also computed the Jaccard index for background (*JI*_*B*_) and foreground (*JI*_*F*_) and their mean (*m*_*IoU*_). Similarly, we computed each model’s dice score coefficient (*DSC*) on the four diverse datasets.

## Results and discussion

In this section, we provide a detailed analysis of the results obtained from our experiments using the proposed method. We discuss the implications of these results and their significance in the context of mitochondrial segmentation. Additionally, we present the comparison of our results with previously published state-of-the-art methods in tabular form for each dataset. This comparative analysis helps evaluate our proposed method’s performance with existing approaches and highlights its strengths and contributions.

### Experimental settings

In our experiments, the training and testing of both the P1 and P2 models were conducted on a desktop environment with comprehensive system specifications: Memory: 64 GB, Processor: Intel Core i7–6700K CPU (4 GHz), Graphics: NVIDIA GeForce GTX TITAN, and OS Type: 64-bit. The Ubuntu 20.04.4 LTS (Focal Fossa) operating system was used to conduct the experiments. We implemented the training and testing codes in the PyTorch framework, leveraging its capabilities for deep learning tasks.

We utilized four publicly available mitochondria datasets during the experiments, as shown in Fig 1. These datasets consist of diverse mitochondria structures, posing a challenge for the model and the proposed fusion method to segment the regions of interest accurately. The variations in mitochondrial structure across the datasets added complexity to the segmentation task. However, our model’s ability to adapt and generalize well to different shapes and textures reassures its robustness.

The Mask R-CNN model parameters, such as learning rate, number of epochs, loss function, etc., were consistent across all datasets during the training and testing phases. Similarly, the 3D U-Net model followed the same parameter settings for each dataset. All four datasets underwent the pre-processing stage in both the training and testing stages, as depicted in Fig 3. This ensured that the input data for both models was prepared and standardized, allowing for fair and consistent comparisons across the datasets.

### Model selection for ensemble method

**Fig 8:**
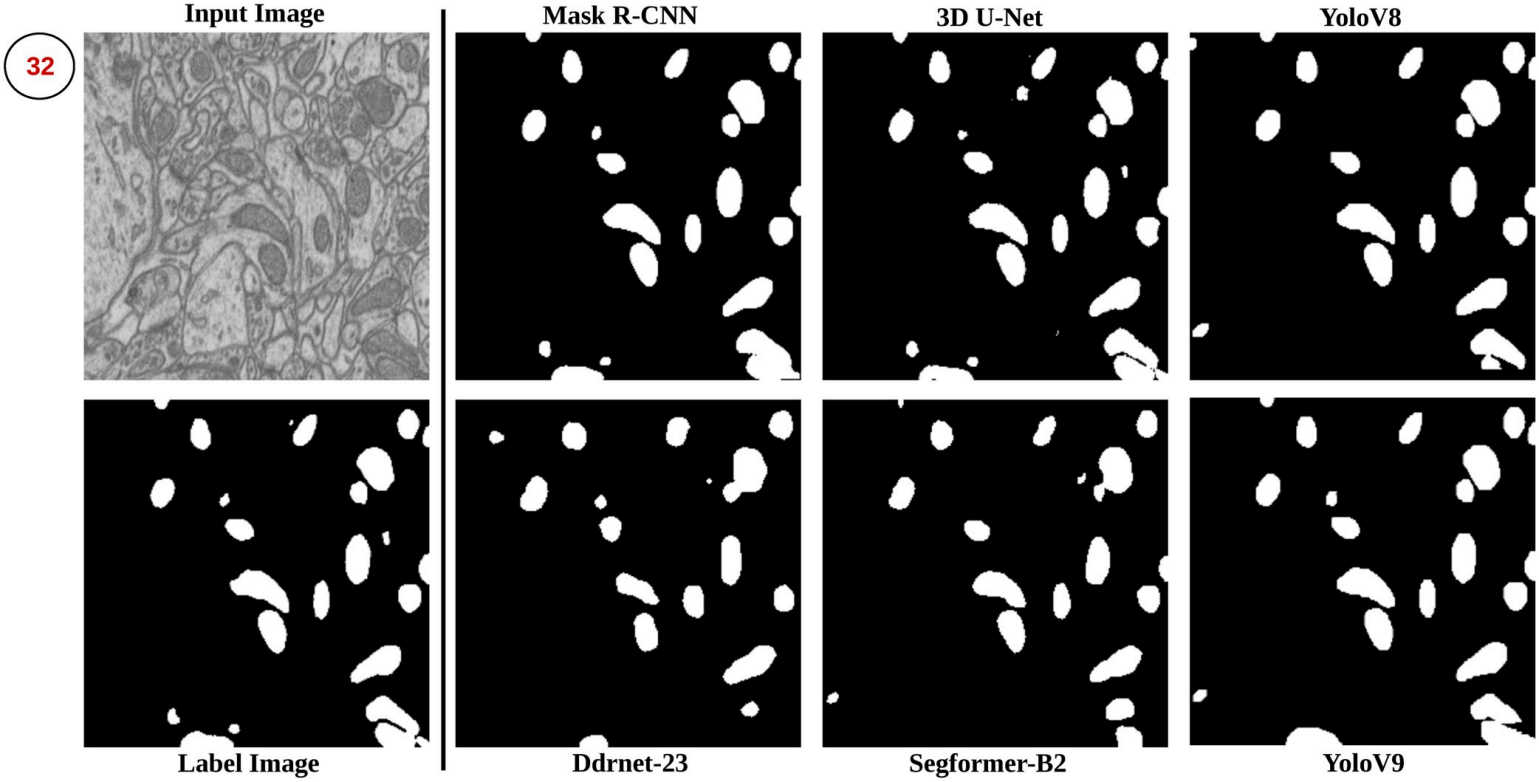
Qualitative comparison of segmentation results across different deep learning models for Lucchi++ slices. The ground truth image of slice 32 is shown alongside predicted segmentations from all six models: Mask R-CNN, 3D U-Net, YoloV8, Ddrnet-23, Segformer-B2, and YoloV9.

**Fig 9:**
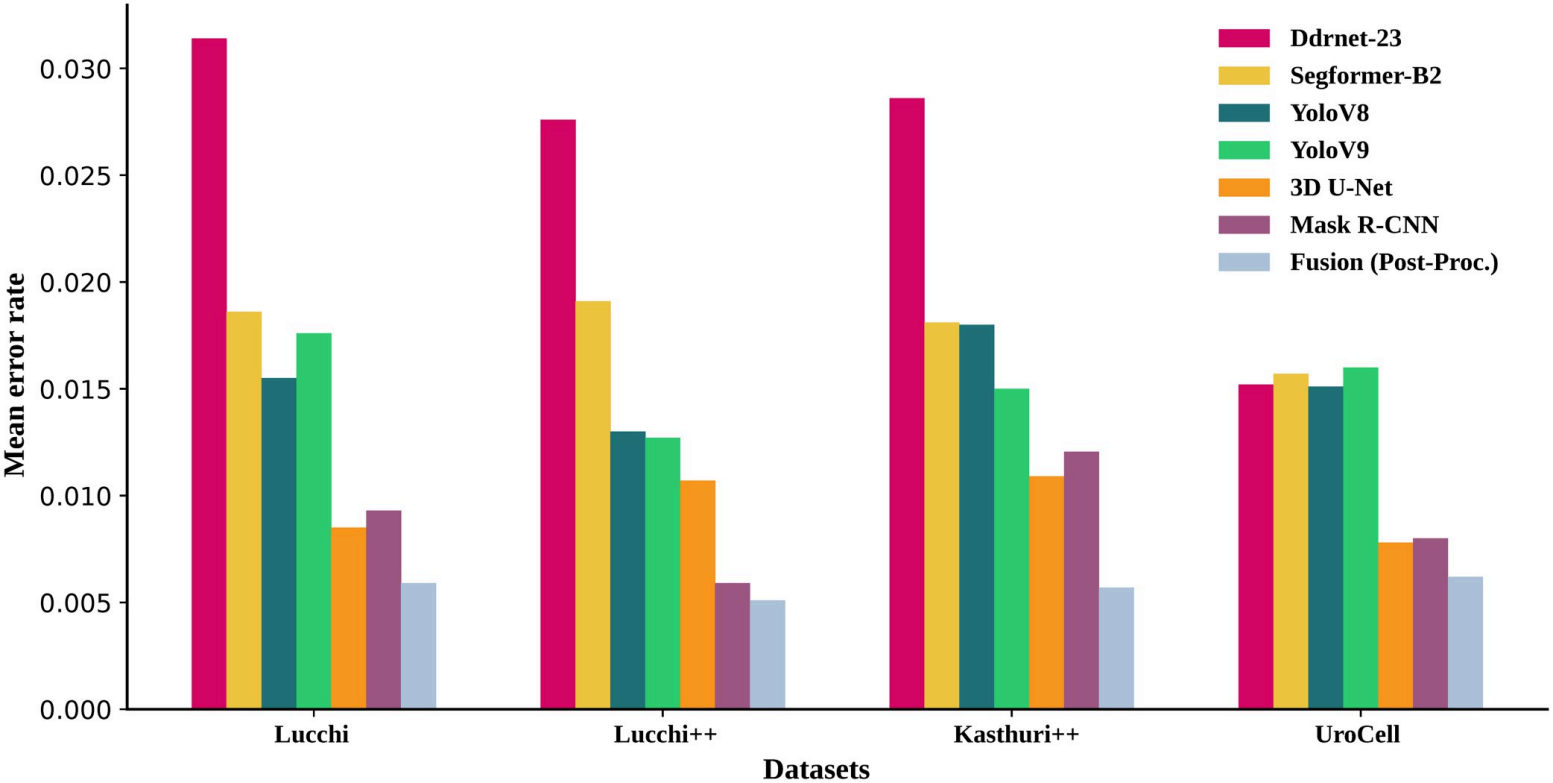
Computed mean error rate for each algorithm and dataset.

Based on our comprehensive evaluation, we selected Mask R-CNN and 3D U-Net for our ensemble method due to their superior performance across various metrics on the four mitochondrial datasets: Lucchi, Lucchi++, Kasthuri++, and UroCell. As depicted in Tables 2 and 3, Mask R-CNN and 3D U-Net consistently achieved higher *JI*_*B*_ and *JI*_*F*_, as well as higher *m*_*IoU*_ and *DSC* values, compared to other models such as Ddrnet-23, Segformer-B2, YoloV8, and YoloV9. Additionally, their AUROC and pixel accuracy metrics were notably better. Fig 8 further illustrates that the segmentations produced by Mask R-CNN and 3D U-Net closely align with the ground truth, demonstrating fewer misclassifications and artifacts. Similarly, Fig 9 shows the obtained error rate for each model, and it further validates that 3D U-Net and Mask R-CNN models have achieved a low error rate compared to the other four models.

The architectural strengths of Mask R-CNN in object detection and 3D U-Net in volumetric segmentation make them particularly effective for detailed and accurate segmentation tasks. In contrast, Ddrnet-23 and Segformer-B2, while competitive, displayed slightly lower performance metrics. Ddrnet-23, which focuses on high-resolution segmentation, struggled with the fine-grained details in mitochondrial structures. Segformer-B2, which combines Transformer and CNN features, showed potential but was less adept at capturing intricate mitochondrial details, leading to lower precision and recall scores. The Yolo models, YoloV8 and YoloV9, primarily designed for real-time object detection, performed well in identifying objects but were less effective in pixel-wise segmentation tasks, as evidenced by their lower mIoU and DSC scores. This is likely due to their optimization for speed and detection rather than the fine-tuned segmentation required for biomedical images. The parameters, such as loss function, optimizer, and learning rate, were consistent while the results were validated.

Thus, the empirical evidence supports our choice of Mask R-CNN and 3D U-Net for the ensemble, leveraging their strengths to achieve more accurate segmentation results. The output variations between models can be attributed to their inherent architectural differences and design perspectives. Mask R-CNN and 3D U-Net are more suited for the detailed and accurate segmentation needed for these datasets.

### Model complexity

The computational complexity of our ensemble approach is primarily characterized by the models’ parameters and GFLOPs (Giga Floating Point Operations per Second), reflecting the scalability and efficiency of the method, as provided in Table 1. The models in our ensemble, such as the 3D U-Net and Mask R-CNN, contain 19 million and 43 million parameters, respectively, highlighting their substantial capacity for detailed feature extraction. The GFLOPs metric further quantifies this computational demand, with values ranging from 17.944 for DDRNet-23 to 344.9 for YoloV8. These values indicate the varying computational costs associated with each model. Specifically, YoloV8, with the highest GFLOPs, provides powerful object detection and segmentation capabilities but requires significant computational resources. In contrast, DDRNet-23, with lower GFLOPs, offers a more computationally efficient option while maintaining robust performance. Our choice of models balances these factors, leveraging the strengths of both high-capacity and efficient models. The consistent use of a batch size of 2 across all models ensures a uniform approach to training. At the same time, the learning rates and optimization strategies (e.g., ADAM and SGD) are tailored to each model’s architecture to optimize convergence and performance. Overall, the ensemble approach strategically integrates diverse models, providing enhanced segmentation accuracy while maintaining feasible computational demands. This balance supports the method’s application to large datasets, as evidenced by the detailed computational costs manageable within current hardware capabilities.

**Table 1:** Summary of model configurations and computational complexities of each model. The GFLOPs metric indicates the computational cost associated with each model, providing insight into their scalability and efficiency.

Models	Batch Size	Learning Rate	Loss Function	Optimize	GFLOPs
**Ddrnet-23** [29]	2	0.0001	BCEDiceLoss	ADAM	17.944
**Segformer-B2** [30]	2	0.0001	BCEDiceLoss	ADAM	60.84
**YoloV8** [31]	2	0.0001	BboxLoss + v8SegmentationLoss	ADAM	344.9
**YoloV9** [32]	2	0.0001	BboxLoss + v8SegmentationLoss	ADAM	159.4
**3D U-Net** [38]	2	0.0002	BCEDiceLoss	ADAM	244.8
**Mask R-CNN** [20]	2	0.005	multi-task loss	SGD	133.9

### Segmentation results

To validate the applicability and effectiveness of our proposed approaches, we conducted experiments on four diverse datasets: Lucchi, Lucchi++, Kasthuri++, and UroCell. Tables 2 and 3 present, respectively, the performance metrics and the computed AUROC, pixel accuracy, and error rates for all four distinct datasets and all three proposed algorithms. Fig 9 presents the mean error rate for each algorithm and dataset. By testing our methods on these datasets, which encompass various mitochondrial structures and imaging conditions, we aimed to assess our approaches’ robustness and generalization capability across various scenarios.

**Table 2:** Performance metrics evaluation of each algorithm on four diverse datasets.

Datasets	Algorithms	JI_F_	JI_B_	m_IoU_	DSC	ACC	PRE	Recall
**Lucchi**	**Ddrnet-23** [29]	0.6365	0.9674	0.7519	0.6952	0.9686	0.748	0.6597
	**Segformer-B2** [30]	0.6877	0.9806	0.8341	0.8135	0.9814	0.9004	0.7482
	**YoloV8** [31]	0.7692	0.9836	0.8764	0.8669	0.9845	0.8071	0.9423
	**YoloV9** [32]	0.7473	0.9815	0.8644	0.8534	0.9824	0.7886	0.9341
	**3D U-Net** [38]	0.912	0.9941	0.9502	0.9512	0.9944	0.9673	0.9503
	**Mask R-CNN** [20]	0.9112	0.9915	0.9512	0.9533	0.9921	0.9416	0.9416
	**Fusion (Post-Proc.)**	**0.9337**	**0.9954**	**0.9623**	**0.9633**	**0.9944**	**0.9761**	**0.9928**
**Lucchi++**	**Ddrnet-23** [29]	0.6955	0.9707	0.8181	0.7977	0.9724	0.8601	0.7496
	**Segformer-B2** [30]	0.7553	0.9796	0.8675	0.86	0.9809	0.92	0.8099
	**YoloV8** [31]	0.838	0.986	0.912	0.9107	0.987	0.8907	0.9347
	**YoloV9** [32]	0.8398	0.9864	0.9131	0.9114	0.9873	0.8718	0.9581
	**3D U-Net** [38]	0.9132	0.9929	0.9537	0.9543	0.9934	**0.9741**	0.9416
	**Mask R-CNN** [20]	0.9302	0.9945	0.9624	0.9649	0.9949	0.9572	0.9705
	**Fusion (Post-Proc.)**	**0.9415**	**0.9952**	**0.9674**	**0.971**	**0.9989**	0.9664	**0.9995**
**Kasthuri++**	**Ddrnet-23** [29]	0.6296	0.9708	0.6752	0.6987	0.9714	0.5282	0.6952
	**Segformer-B2** [30]	0.6914	0.9856	0.8035	0.7658	0.9859	0.7763	0.7593
	**YoloV8** [31]	0.7103	0.9847	0.7731	0.7671	0.9645	0.755	0.7493
	**YoloV9** [32]	0.7222	0.9899	0.8009	0.8142	0.9872	0.847	0.775
	**3D U-Net** [38]	0.9175	0.9914	0.9518	0.9512	0.992	0.9557	0.9464
	**Mask R-CNN** [20]	0.8823	0.9974	0.9499	0.9375	0.9974	0.9585	0.9173
	**Fusion (Post-Proc.)**	**0.9441**	**0.9982**	**0.9711**	**0.9712**	**0.9982**	**0.9936**	**0.9599**
**UroCell**	**Ddrnet-23** [29]	0.8677	0.9941	0.9309	0.9287	0.9943	0.9415	0.9175
	**Segformer-B2** [30]	0.8772	0.9946	0.9359	0.9335	0.9948	0.9585	0.9117
	**YoloV8** [31]	0.8437	0.9926	0.9181	0.9122	0.9929	0.9291	0.9023
	**YoloV9** [32]	0.8566	0.9937	0.9252	0.9217	0.994	0.9712	0.8792
	**3D U-Net** [38]	0.9329	0.9949	0.9601	0.9622	0.9952	0.9344	0.9620
	**Mask R-CNN** [20]	0.9231	0.9951	0.9623	0.9654	0.9954	0.9579	0.9579
	**Fusion (Post-Proc.)**	**0.9432**	**0.9956**	**0.9644**	**0.9749**	**0.9958**	**0.9689**	**0.9895**

ACC: Accuracy, PRE: Precision

**Table 3:** Computed AUROC and pixel accuracy for all four distinct datasets using all seven proposed algorithms.

Datasets	Algorithms	AUROC	Pixel Accuracy
**Lucchi**	**Ddrnet-23** [29]	0.8236	0.9686
	**Segformer-B2** [30]	0.8718	0.9814
	**YoloV8** [31]	0.9646	0.9845
	**YoloV9** [32]	0.9597	0.9824
	**3D U-Net** [38]	0.9712	0.9915
	**Mask R-CNN** [20]	0.9677	0.9907
	**Fusion (Post-Proc.)**	**0.9937**	**0.9941**
**Lucchi++**	**Ddrnet-23** [29]	0.8704	0.9724
	**Segformer-B2** [30]	0.9024	0.9809
	**YoloV8** [31]	0.9632	0.987
	**YoloV9** [32]	0.9738	0.9873
	**3D U-Net** [38]	0.9695	0.9793
	**Mask R-CNN** [20]	0.9837	0.9841
	**Fusion (Post-Proc.)**	**0.9931**	**0.9949**
**Kasthuri++**	**Ddrnet-23** [29]	0.7794	0.9714
	**Segformer-B2** [30]	0.8761	0.9859
	**YoloV8** [31]	0.874	0.9814
	**YoloV9** [32]	0.9112	0.9864
	**3D U-Net** [38]	0.9713	0.9945
	**Mask R-CNN** [20]	0.9583	0.9891
	**Fusion (Post-Proc.)**	**0.9748**	**0.9983**
**UroCell**	**Ddrnet-23** [29]	0.9575	0.9943
	**Segformer-B2** [30]	0.955	0.9948
	**YoloV8** [31]	0.9495	0.9929
	**YoloV9** [32]	0.939	0.994
	**3D U-Net** [38]	0.9825	0.9922
	**Mask R-CNN** [20]	0.9717	0.9888
	**Fusion (Post-Proc.)**	**0.9985**	**0.9938**

**Fig 10:**
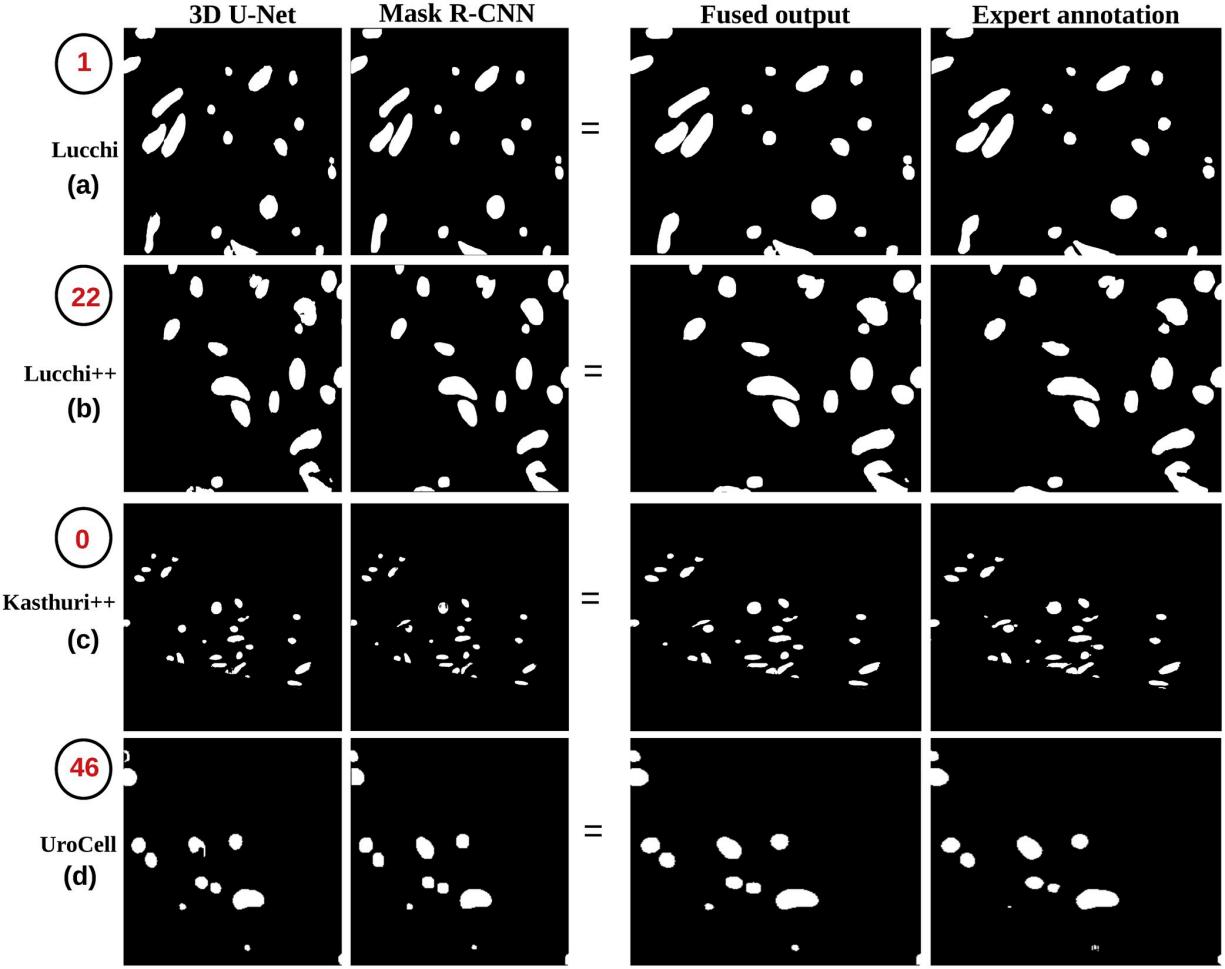
The figure displays the final segmentation results achieved by applying the ensemble learning weight average fusion technique across four datasets, presented alongside their respective annotated label images. The black circle indicates the slice number of each image. The fused output highlights the advantages of the weight fusion technique, enhancing segmentation accuracy by combining the strengths of Mask R-CNN and 3D U-Net predictions.

Our fusion method consistently outperforms the Ddrnet-23, Segformer-B2, YoloV8, and YoloV9, Mask R-CNN, and 3D U-Net model (Fig 10) and other state-of-the-art methods regarding foreground segmentation accuracy as shown in Tables 4–7. This indicates that the fusion of predictions from multiple models enhances the overall performance and leads to more accurate and robust segmentation results.

**Table 4:** Performance comparison of segmentation methods on the Lucchi dataset.

Methods	JI_F_	m_IoU_	DSC
2D U-Net without z-filtering [21]	0.878	0.935	NA
2D U-Net with z-filtering [21]	0.89	0.942	NA
Sliding window CNN + post-proc. [17]	0.907	NA	NA
3D U-Net + Res. Blocks [2]	0.9	NA	NA
3D U-Net [42]	0.889	0.942	NA
3D U-Net [2]	0.874	NA	NA
2D U-Net [42]	0.865	0.928	NA
PIBoost (multi-class boosting) [43]	0.76	NA	NA
Random fields [44]	0.762	NA	NA
nnU-Net framework [45]	0.882	0.938	NA
2D Residual U-Net [37]	0.885	0.939	NA
2D U-Net [37]	0.893	0.942	NA
2D Attention U-Net [37]	0.893	0.943	NA
3D U-Net [37]	0.885	0.939	NA
3D Attention U-Net [37]	0.88	0.936	NA
3D Residual U-Net [37]	0.888	0.941	NA
LUCCHI-TMI [33]	NA	0.84	NA
LUCCHI-CVPR [8]	0.755	0.868	0.86
LUCCHI-MICCAI [46]	0.741	NA	0.851
HIVE-Net (single-task) [24]	0.892	NA	0.943
2D segmentation using single view [11]	0.764	0.875	0.866
Final multi-view fusion [11]	0.785	0.893	0.879
Hierarchical multi-view fusion [11]	0.826	0.908	0.905
3D segmentation with median fusion [11]	0.833	0.909	0.909
3D segmentation with adaptive template transformer [27]	0.902	NA	0.948
3D segmentation with coherent fragment vision transformer [28]	0.905	NA	0.95
**Ddrnet-23** [29]	0.6365	0.7519	0.6952
**Segformer-B2** [30]	0.6877	0.8341	0.8135
**YoloV8** [31]	0.7692	0.8764	0.8669
**YoloV9** [32]	0.7473	0.8644	0.8534
**3D U-Net** [38]	0.912	0.9502	0.9512
**Mask R-CNN** [20]	0.9112	0.9512	0.9533
**Fusion (Post-Proc.)**	**0.9337**	**0.9623**	**0.9633**

**Table 5:** Performance comparison of segmentation methods on the Lucchi++ dataset.

Methods	JI_F_	m_IoU_	DSC
2D U-Net without z-filtering [21]	0.888	0.94	NA
2D U-Net with z-filtering [21]	0.9	0.946	NA
2D Residual U-Net* [37]	0.908	0.943	NA
2D U-Net* [37]	0.916	0.955	NA
2D Attention U-Net* [37]	0.919	0.956	NA
3D U-Net** [37]	0.923	0.958	NA
3D Attention U-Net** [37]	0.923	0.959	NA
3D Residual U-Net** [37]	0.926	0.96	NA
Strategy One (Multi-cue for seg.) [47]	0.908	0.948	0.949
Strategy Two (Median filter) [47]	0.868	0.929	0.93
Strategy Three*** [47]	0.93	0.963	0.964
Strategy Three**** [47]	0.935	0.965	0.966
**Ddrnet-23** [29]	0.6955	0.8181	0.7977
**Segformer-B2** [30]	0.7553	0.8675	0.86
**YoloV8** [31]	0.838	0.912	0.9107
**YoloV9** [32]	0.8398	0.9131	0.9114
**3D U-Net** [38]	0.9132	0.9537	0.9543
**Mask R-CNN** [20]	0.9302	0.9624	0.9649
**Fusion (Post-Proc.)**	**0.9415**	**0.9674**	**0.971**

(*) blended ensemble and z-filtering post-processing,

(**) ensemble post-processing,

(***) (Multi-slice fusion) without TTA,

(****) (Multi-slice fusion) + TTA 3

**Table 6:** Performance comparison of segmentation methods on the Kasthuri++ dataset.

Methods	JI_F_	m_IoU_	DSC
2D U-Net without z-filtering [21]	0.845	0.92	NA
2D U-Net with z-filtering [21]	0.846	0.92	NA
2D Residual U-Net* [37]	0.908	0.953	NA
2D U-Net* [37]	0.915	0.956	NA
2D Attention U-Net* [37]	0.916	0.955	NA
3D U-Net* [37]	0.934	0.965	NA
3D Attention U-Net* [37]	0.934	0.966	NA
3D Residual U-Net* [37]	0.937	0.967	NA
LUCHI-CVPR [8]	0.758	0.875	0.862
HIVE-Net (single-task) [24]	0.917	NA	0.957
2D segmentation using single view [11]	0.772	0.882	0.871
Hierarchical multi-view fusion [11]	0.814	0.904	0.897
3D segmentation with median fusion [11]	0.806	0.9	0.893
**Ddrnet-23** [29]	0.6296	0.6752	0.6987
**Segformer-B2** [30]	0.6914	0.8035	0.7658
**YoloV8** [31]	0.7103	0.7731	0.7671
**YoloV9** [32]	0.7222	0.8009	0.8142
**3D U-Net** [38]	0.9175	0.9518	0.9512
**Mask R-CNN** [20]	0.8823	0.9499	0.9375
**Fusion (Post-Proc.)**	**0.9441**	**0.9711**	**0.9712**

(*) ensemble post-processing

**Table 7:** Performance comparison of segmentation methods on the UroCell dataset.

Methods	JI_F_	m_IoU_	DSC	TPR	TNR
U-Net [5]	NA	NA	0.855	0.825	0.997
V-Net [5]	NA	NA	0.898	0.873	**0.999**
DeepMedic [5]	NA	NA	0.867	0.856	0.997
HighRes3DNet [5]	NA	NA	0.883	0.857	**0.999**
HighRes3DNet* [5]	NA	NA	0.903	0.881	**0.999**
HighRes3DNet** [5]	NA	NA	0.954	0.942	**0.999**
HighRes3DZMNet [5]	NA	NA	0.882	0.852	**0.999**
HighRes3DZMNet** [5]	NA	NA	0.937	0.921	**0.999**
HighRes3DZMNet*** [5]	NA	NA	0.939	0.927	**0.999**
HighRes3DZMNet**** [5]	NA	NA	0.942	0.921	**0.999**
**Ddrnet-23** [29]	0.8677	0.9309	0.9287	0.9175	0.9975
**Segformer-B2** [30]	0.8772	0.9359	0.9335	0.9117	0.9983
**YoloV8** [31]	0.8437	0.9181	0.9122	0.9023	0.9967
**YoloV9** [32]	0.8566	0.9252	0.9217	0.8792	0.9988
**3D U-Net** [38]	0.9329	0.9601	0.9622	0.9620	0.9980
**Mask R-CNN** [20]	0.9231	0.9623	0.9654	0.9579	0.9975
**Fusion (Post-Proc.)**	**0.9432**	**0.9644**	**0.9749**	**0.9895**	0.9975

(*) with transfer learning (TRAN),

(**) with contrast enhancement (CON),

(***) with CON and segmentation masks,

(****) only upper branch

#### Lucchi dataset

On the testing data, our proposed fusion method achieved an accuracy of 0.9337, 0.9954, 0.9623, 0.9633, 0.9944, 0.9761, and 0.9928 scores in terms of *JI*_*F*_, *JI*_*B*_, *m*_*IoU*_, *DSC*, accuracy, precision, and recall, respectively, as shown in Table 2. Likewise, in the case of the 3D U-Net model (which we used from [38]), which is a pixel-wise segmentation method designed to track the 3D connectivity of volumes in 3D data, the performance of the 3D U-Net model on this dataset was slightly lower than the fusion method, achieving scores of 0.912, 0.9941, 0.9502, 0.9512, 0.9944, 0.9673, and 0.9503 for *JI*_*F*_, *JI*_*B*_, *m*_*IoU*_, *DSC*, accuracy, precision, and recall, respectively, as shown in Table 2.

**Fig 11:**
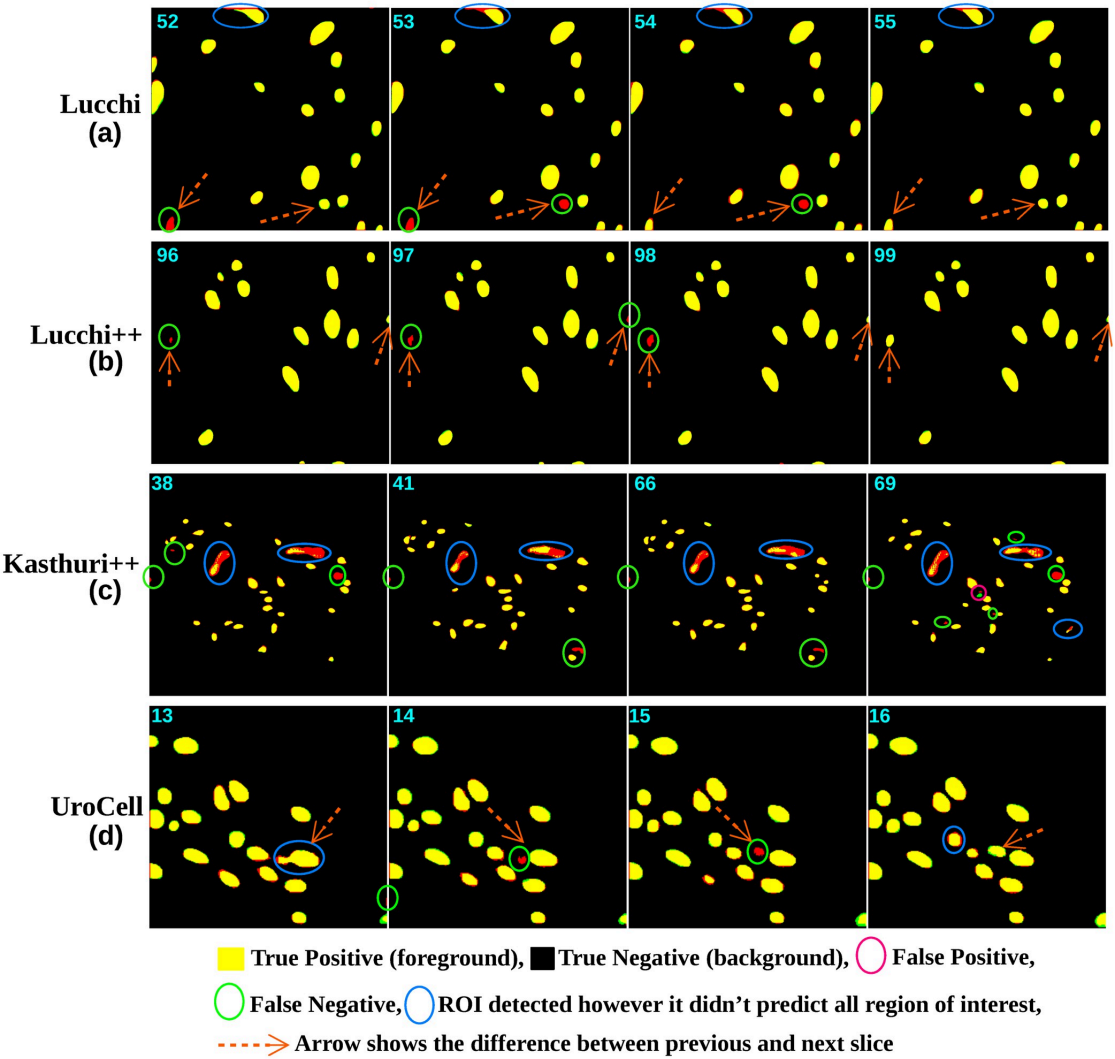
The image illustrates the predicted output of Mask R-CNN (green channel) overlapped with the ground-truth image (red channel) for various datasets. The yellow-colored regions represent true positive regions where Mask R-CNN accurately segmented the interested regions of interest (ROI). However, limitations of the model can also be observed. For example, in the Lucchi dataset, a particular object was not detected in slice numbers 52 and 53 (indicated by the orange arrow) but detected in slices 54 and 55, highlighting challenges in object localization for connected objects. Similar limitations can be seen in the Lucchi++ dataset. In the case of the Kasturi++ dataset, while most objects were correctly detected, there are instances of false negative ROIs (indicated by the green circle). The blue circle highlights regions where the model predicted correctly, but the segmentation did not fully cover the entire ROI.

**Fig 12:**
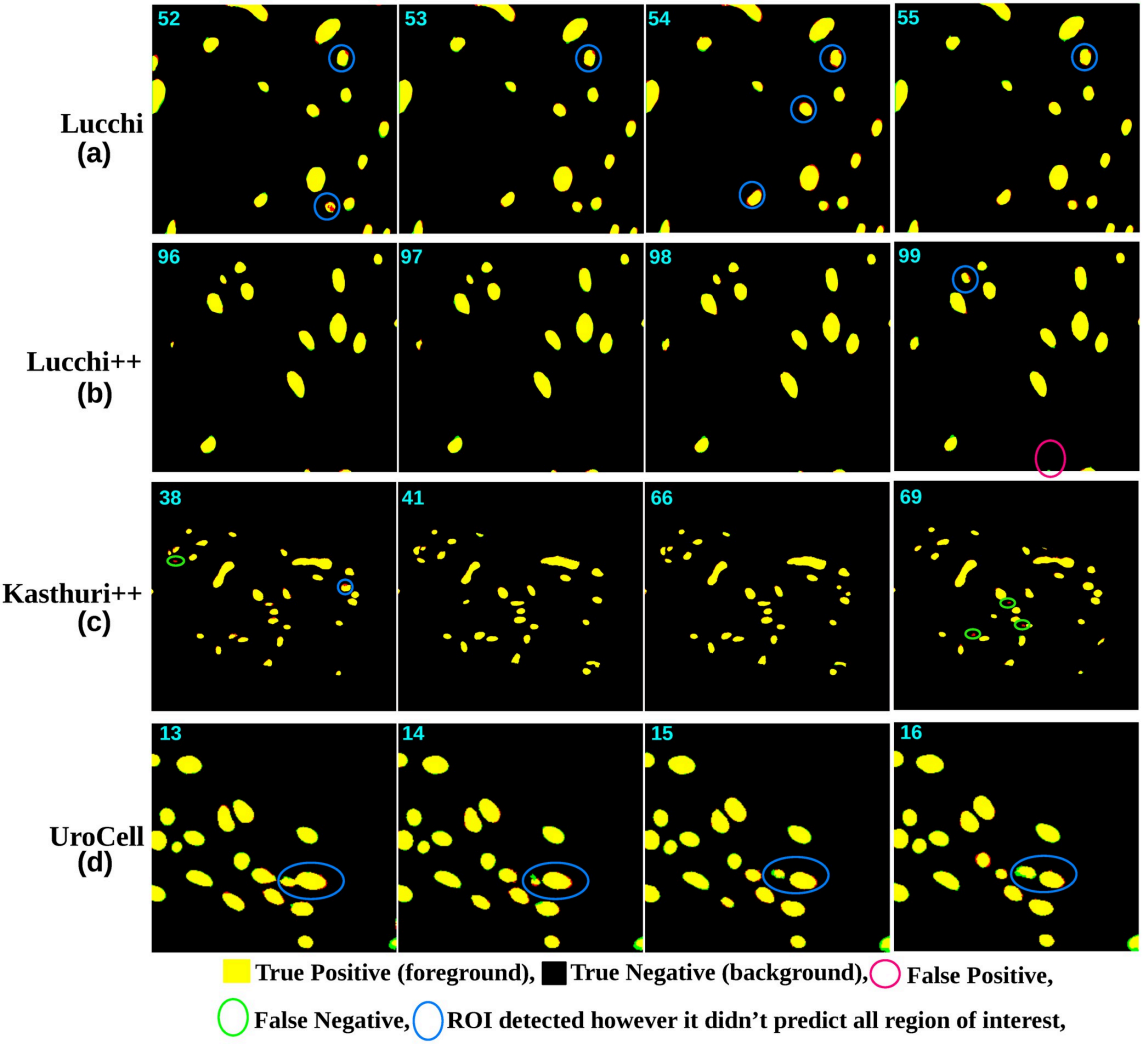
The image displays the comparison between the predicted output of the 3D U-Net model (shown in the green channel) and the ground-truth image (shown in the red channel) for various datasets. The yellow-colored regions indicate the true positive regions where the 3D U-Net model accurately segmented most ROIs. Overall, the performance of the 3D U-Net model is relatively good compared to the predictions from Mask R-CNN. However, there are still instances, such as in slice number 52, where the 3D U-Net model did not fully predict certain objects. An example of such an object is highlighted with a blue circle, indicating that it was not completely captured by the trained model (M2). The green circle shows the false negative ROIs, which should have been predicted, but the model failed to predict it.

Despite this, the 3D U-Net model still demonstrated exemplary performance in accurately segmenting the mitochondria regions of interest, exhibiting its effectiveness in capturing the 3D connectivity of the structures, which can be seen in Fig 12a. The performance of the Mask R-CNN method on this dataset was relatively average compared to the earlier two methods. The scores obtained for *JI*_*F*_, *JI*_*B*_, *m*_*IoU*_, *DSC*, accuracy, precision, and recall were 0.9112, 0.9915, 0.9512, 0.9533, 0.9921, 0.9416, and 0.9416, respectively, as shown in Table 2. One possible reason for this average performance could be the limitation of the two-dimensional object localization technique used in Mask R-CNN when dealing with 3D-connected regions. The method may struggle to accurately track and localize these connected components, leading to localization problems. This can be observed in Fig 11a, where some 3D connected objects are not precisely localized or localized in the first slice but did not localize in the second or fourth slice.

Likewise, in Table 3, we have computed the AUROC, pixel accuracy, and error rate for each algorithm. It is evident from this table that the fusion algorithm has achieved impressive scores, with 0.9937 and 0.9941 for AUROC and pixel accuracy, respectively. These scores are notably higher than those obtained by the 3D U-Net and Mask R-CNN algorithms. Furthermore, in terms of the mean error rate, the fusion method stands out as the best performer, with a mean error rate of 0.0059 (Fig 9). In comparison, the 3D U-Net has a mean error rate of 0.0085, and Mask R-CNN has a mean error rate of 0.0093. Table 4 provides a comprehensive evaluation of our proposed fusion method, offering a detailed comparison with the results obtained by other authors on the same dataset. The table shows that our proposed fusion method has obtained competitive scores across all the listed metrics compared to other methods.

#### Lucchi++ dataset

In the case of the Lucchi++ testing data, our proposed fusion method achieved scores of 0.9415, 0.9952, 0.9674, 0.971, 0.9989, 0.9664, and 0.9995 for *JI*_*F*_, *JI*_*B*_, *m*_*IoU*_, *DSC*, accuracy, precision, and recall, respectively (as detailed in Table 2). The fusion method demonstrated strong performance in accurately segmenting this dataset’s mitochondria regions of interest. Similarly, the Mask R-CNN model also performed well for this data, achieving scores of 0.9302, 0.9945, 0.9624, 0.9649, 0.9949, 0.9572, and 0.9705 for *JI*_*F*_, *JI*_*B*_, *m*_*IoU*_, *DSC*, accuracy, precision, and recall, respectively. The performance of the Mask R-CNN model was comparable to the fusion method, exhibiting its effectiveness in segmenting the mitochondria structures despite having some limitations in detecting 3D-connected objects. The 3D U-Net model achieved scores of 0.9132, 0.9929, 0.9537, 0.9543, 0.9934, 0.9741, and 0.9416 for *JI*_*F*_, *JI*_*B*_, *m*_*IoU*_, *DSC*, accuracy, precision, and recall, respectively, for this dataset. Although scores are slightly lower than the fusion method and Mask R-CNN, the 3D U-Net model still performed well in segmenting the mitochondria regions of interest.

Overall, the results obtained from the Lucchi++ dataset validate the applicability and effectiveness of our proposed approaches in accurately segmenting diverse mitochondria structures. Figs 11b and 12b provide a visual comparison of the performance of the Mask R-CNN and 3D U-Net models on slices from 96 to 99. In the case of the Mask R-CNN results, some false negatives were observed, and it is also evident that an object that was not detected in slice 96 is later detected in slice 99, highlighting the limitations of object detection algorithms. On the other hand, the 3D U-Net model performs well on these slices, with only one false positive observed in slice 99 and false negatives in the top corner object of the same slice.

Similarly, Table 3 displays each algorithm’s AUROC, pixel accuracy, and mean error rate values. The fusion method stands out with impressive scores: 0.9931 for AUROC, 0.9949 for pixel accuracy, and a low mean error rate of 0.0051. These results are notably superior to the performance of the 3D U-Net and Mask R-CNN algorithms. Table 5 provides a comprehensive comparison of the results obtained by other authors on the Lucchi++ dataset, offering insights into the effectiveness of our proposed fusion method. In terms of *JI*_*F*_ and *DSC* scores, the table demonstrates that our proposed fusion method has achieved competitive results compared to other methods. However, in terms of *m*_*IoU*_, the proposed pipeline by [47] strategy three (multi-slice fusion) with TTA 3 achieved score (0.965) is competitive to our proposed fusion method.

#### Kasthuri++ dataset

The results, shown in Table 2, indicate that our proposed fusion method achieved impressive scores across all seven metrics: *JI*_*F*_ (0.9441), *JI*_*B*_ (0.9982), *m*_*IoU*_ (0.9711), *DSC* (0.9712), accuracy (0.9982), precision (0.9936), and recall (0.9599).

In the case of Mask R-CNN, the obtained results for *JI*_*F*_ are not competitive compared to the scores achieved by the proposed fusion method or other authors. For example, the several variants of the 2D and 3D U-Net pipeline proposed by [37] have outperformed the Mask R-CNN scores in terms of *JI*_*F*_ and *m*_*IoU*_. Fig 11c highlights the limitations of the Mask R-CNN model, as it exhibits numerous false negatives in the predicted results. Despite partially detecting some regions of the false negatives, Mask R-CNN fails to accurately segment the complete objects, as indicated by the blue circle in the figure. Additionally, there are instances of false positive predicted ROIs, which are indicated by the pink circle in the same figure. Similarly, the performance of the 3D U-Net model on the Kasthuri++ dataset was slightly lower when compared to our proposed fusion method and the several variants of the 2D and 3D U-Net proposed pipeline by [37]. The obtained scores for *JI*_*F*_, *JI*_*B*_, *m*_*IoU*_, *DSC*, accuracy, precision, and recall were 0.9175, 0.9914, 0.9518, 0.9512, 0.992, 0.9557, and 0.9464, respectively, as shown in Table 2. Fig 12c presents the predicted results of the 3D U-Net model on slices 38, 41, 66, and 69. The model demonstrates its capability to accurately segment most objects in the images, compared to the predicted results of Mask R-CNN shown in Fig 11c. This suggests that the 3D U-Net model can effectively capture and delineate the structures of interest in the Kasthuri++ dataset.

Likewise, Table 3 summarizes the AUROC, pixel accuracy, and mean error rate values for each algorithm on the Kasthuri++ dataset. The fusion method excels with impressive scores: 0.9748 for AUROC, 0.9983 for pixel accuracy, and a low mean error rate of 0.0057. These results significantly outperform the performance of the 3D U-Net and Mask R-CNN algorithms.

#### UroCell dataset

In the case of the UroCell dataset, which is a newly available dataset, our proposed ensemble learning-based weight average fusion method, as well as the Mask R-CNN and 3D U-Net models, performed exceptionally well compared to the methods proposed by [5]. The proposed fusion method achieved high scores in all seven metrics, with scores of 0.9432, 0.9956, 0.9644, 0.9749, 0.9958, 0.9689, and 0.9895 for *JI*_*F*_, *JI*_*B*_, *m*_*IoU*_, *DSC*, accuracy, precision, and recall, respectively, as shown in Table 2. The 3D U-Net model also performed well, with scores of 0.9329, 0.9949, 0.9601, 0.9622, 0.9952, 0.9344, and 0.9622 for the same metrics. The Mask R-CNN model achieved scores of 0.9231, 0.9951, 0.9623, 0.9654, 0.9954, 0.9579, and 0.9579 for *JI*_*F*_, *JI*_*B*_, *m*_*IoU*_, *DSC*, accuracy, precision, and recall, respectively, on the UroCell dataset. These results demonstrate the effectiveness of our proposed fusion method and the individual models in accurately segmenting the UroCell dataset.

Figs 11d and 12d demonstrate the performance of the Mask R-CNN and 3D U-Net models on the UroCell testing volume slices from 13 to 16. Both models performed well on this dataset, with the Mask R-CNN model missing some objects (false negatives) in slices 14 and 15 but successfully predicting the same region in slice 16. This limitation is attributed to the object localization technique’s difficulty segmenting 3D-connected regions. On the other hand, the 3D U-Net model accurately predicted all objects, including the previously missed false negative object, by the Mask R-CNN model.

Similarly, we computed AUROC, pixel accuracy, and mean error rate for each algorithm on this dataset, and the scores are presented in Table 3. Our proposed fusion method achieved impressive scores of 0.9985, 0.9938, and 0.0062 for AUROC, pixel accuracy, and mean error rate, respectively. In comparison, 3D U-Net obtained scores of 0.9825, 0.9922, and 0.0078 for the same metrics, while Mask R-CNN scored 0.9717, 0.9888, and 0.008. These results highlight the superior performance of the fusion method. Additionally, Fig 9 displays the mean error rate for each algorithm across all datasets.

**Fig 13:**
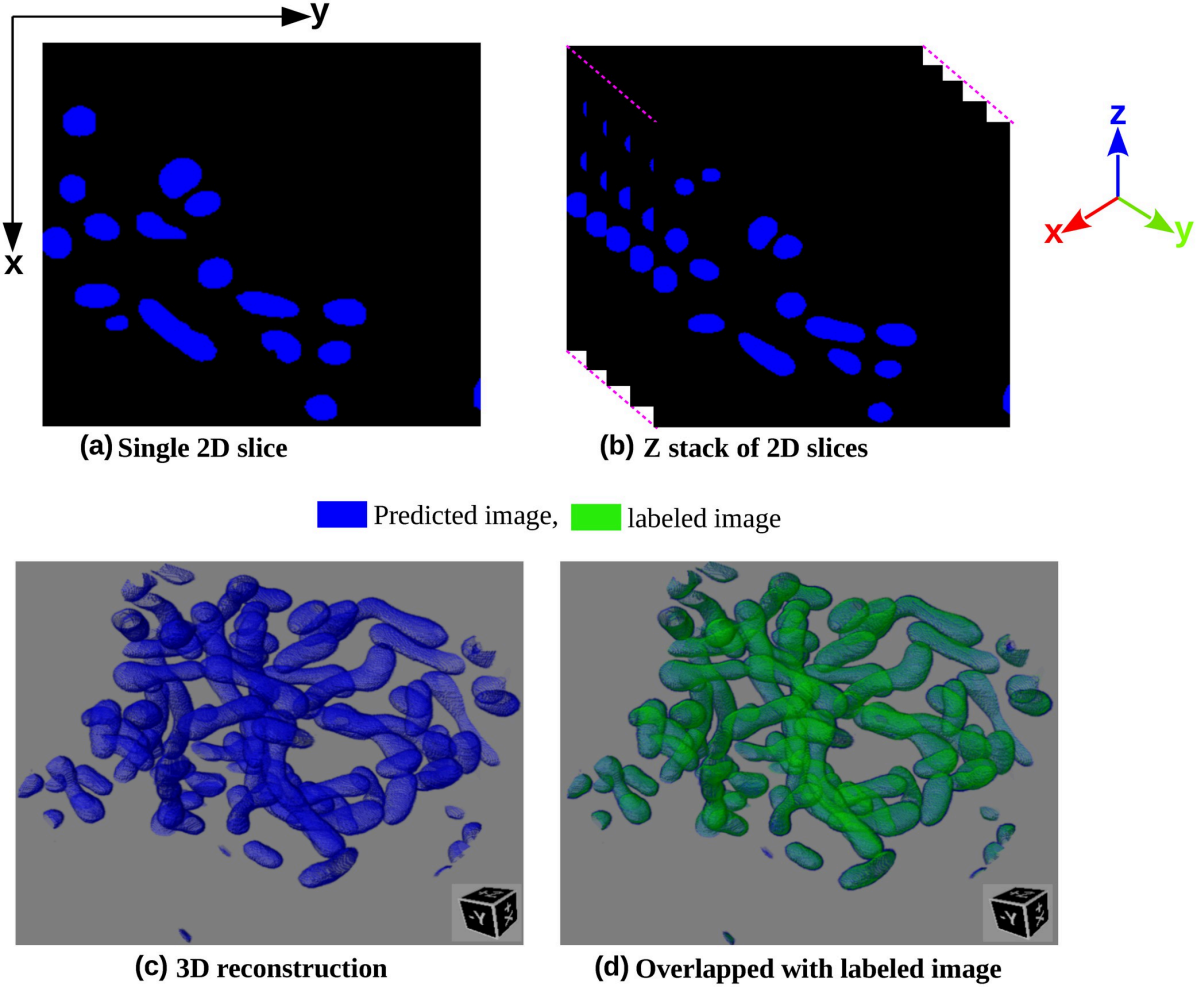
The image shows the visual representation of the segmented UroCell testing dataset by applying the fusion method in (a) 2D, in (b) z-stack, in (c) 3D reconstruction, and in (d) shows the overlapped of predicted volume (shown in blue) with expert annotated ground truth volume of the same testing dataset (shown in green), to see the 3D binding between these two.

Fig 13a and 13b showcase the predicted outputs of the proposed fusion method in 2D and z-stack form, respectively. The predicted slices are highlighted in blue color. Additionally, Fig 13c visualizes the same z-stack predicted slices in 3D using the visualization tool proposed by Gupta et al. [48]. These figures assess whether our proposed fusion method accurately captures the relevant objects in their 3D shapes. By examining Fig 13c, it becomes evident that our proposed method successfully segments all the regions of interest. The 3D connectivity between the slices is blue, indicating accurate segmentation. Furthermore, we overlay the predicted output (shown in blue) with the expert-annotated ground truth (shown in green) to assess the consistency between the proposed fusion segmentation result and the labeled image. Remarkably, the predicted output aligns remarkably well with the labeled image Fig 13d, further confirming the effectiveness and accuracy of our proposed fusion method.

#### Comparison with state-of-the-art methods

In this experiment, we compared our proposed fusion method with several state-of-the-art approaches for mitochondrial segmentation on each dataset. The compared methods include both hand-crafted feature-based methods and deep learning methods, selected based on their reported performance on the specific datasets.

For each dataset, as shown in Tables 4–7, we observed that variants of 2D and 3D U-Net methods, such as residual U-Net and attention U-Net [2, 5, 21, 37, 47, 49], have achieved good performance on segmentation, and their scores are competitive. These variants of U-Net methods aim to enhance the performance of the baseline U-Net architecture by incorporating residual connections and attention mechanisms. Furthermore, some state-of-the-art approaches explored the use of hand-crafted features combined with traditional machine learning techniques, such as random forests, multi-class boosting, and gradient descent methods [8, 11, 43, 44]. These methods often involve manual feature engineering, where domain knowledge and expert input are used to design effective features for mitochondrial segmentation.

For the Lucchi dataset, most state-of-the-art methods achieved scores in the range of 0.85 to 0.90 in terms of *JI*_*F*_, slightly lower than the score obtained by our proposed fusion method, which is 0.9337. One notable method by [17] proposed a sliding window method based on CNN with post-processing, which achieved a performance of 0.907 in terms of *JI*_*F*_, demonstrating relatively high accuracy. However, our proposed fusion method outperformed this approach, showcasing its segmentation accuracy and robustness superiority.

Similarly, in the case of the Lucchi++ dataset, [37] proposed several variants of the 2D and 3D U-Net pipeline, including multi-slice fusion and test-time augmentation (TTA) strategies by [47], which achieved good performance in segmenting mitochondrial regions for this dataset. These approaches demonstrated impressive results regarding the Jaccard Index foreground, mean Intersection over Union, and dice score coefficient, as shown in Table 5. However, our fusion method surpassed their performance, demonstrating its effectiveness in handling the challenges posed by the dataset and providing more accurate segmentation results.

For the Kasthuri++ dataset, [37] utilized deep learning methods such as 3D U-Net (ensemble post-processing), 3D Attention U-Net (ensemble post-processing), and 3D Residual U-Net (ensemble post-processing) to segment the mitochondria. Their approach achieved 0.934, 0.934, and 0.937 scores regarding the *JI*_*F*_. Additionally, [11] employed the extraction of local patch pattern (LPP) features from the images and utilized traditional machine learning techniques such as random forests for prediction. However, the achieved scores were comparatively lower than other state-of-the-art methods that used deep learning approaches for mitochondria segmentation on this dataset. In this case, our proposed fusion method has performed very well while segmenting the diverse structure of mitochondria, highlighting the advantage of leveraging ensemble learning and the combination of Mask R-CNN and 3D U-Net.

For the UroCell dataset, [5] introduced the HighRes3DZMNet algorithm, a modification of the HighRes3DNet model [25], designed explicitly for mitochondria segmentation on this dataset. They explored various combinations of their proposed method with other techniques, such as transfer learning (TRAN), contrast enhancement (CON), and segmentation masks, focusing on the upper branch of their approach. Overall, their HighRes3DNet with contrast enhancement (CON) approach demonstrated good performance, achieving scores of 0.954, 0.942, and 0.999 for *DSC*, true positive rate (*TPR*), and true negative rate (*TNR*), respectively. However, our proposed fusion method surpassed their performance, indicating the strength of our approach in accurately capturing the intricate structures of mitochondria. Our fusion method achieved scores of 0.9749, 0.9895, and 0.9975 for *DSC*, *TPR*, and *TNR*, respectively, showcasing its superiority in segmenting mitochondria on the UroCell dataset.

We computed AUROC, pixel accuracy, and mean error rate for each algorithm across all datasets, and the results are presented in Table 3. Our fusion method consistently achieved the lowest mean error rate, demonstrating superior performance.

The entropy-weighted fusion strategy efficiently integrates Mask R-CNN and 3D U-Net models’ predictions. Utilizing vectorized operations and GPU acceleration, our method maintains computational efficiency while ensuring accurate segmentation. The additional computational overhead is minimal, constituting only a small fraction of total processing time, thus making it practical for real-world applications.

Overall, our fusion method outperformed state-of-the-art methods in segmentation accuracy and robustness. By leveraging the strengths of Mask R-CNN and 3D U-Net models through entropy-weighted average linear fusion, our method effectively overcomes individual model limitations, achieving superior performance in segmenting mitochondria across diverse datasets.

### Limitations

While our proposed entropy-weighted ensemble approach combining Mask R-CNN and 3D U-Net has demonstrated significant improvements in mitochondrial segmentation, as shown in Fig 10, several limitations must be acknowledged, which we found while experimenting:

1. **Handling Diverse Mitochondrial Structures**:
  - Structural Variability: Mitochondria exhibit a wide range of shapes and sizes, varying significantly across different cell types and imaging conditions, as shown in Fig 1. While our method performs well on the datasets used, it may face challenges in generalizing to other datasets with highly irregular or uncommon mitochondrial structures. Future work could explore incorporating additional models specialized in capturing such variability or using more advanced data augmentation techniques to improve generalization.
2. **Imaging Conditions**:
  - Noise and Artifacts: FIB-SEM images often contain noise and artifacts that can impact segmentation accuracy. Although our preprocessing steps aim to mitigate these issues by applying the Gaussian blur method (as shown in Fig 3c), certain types of noise and imaging artifacts may still pose challenges. Advanced noise reduction algorithms and artifact correction techniques could be integrated into the preprocessing pipeline to enhance robustness.
  - Contrast Variability: Variations in image contrast across different datasets can affect model performance, and therefore, in our pipeline, we have applied CLAHE (as shown in Fig 3b image contrast enhancement technique to enhance the contrast of the image, however, same clip-limit might not be suitable for all datasets; therefore, proposed method may require fine-tuning of parameters or additional contrast normalization steps to handle varying imaging conditions effectively.
3. **Post-Processing Strategy**:
  - Entropy-Weighted Fusion: While the entropy-weighted fusion strategy enhances segmentation accuracy by leveraging model confidence (as shown in Tables 4–7), it may not always perfectly capture the nuances of every mitochondrial structure. In some cases, misclassifications from one model might still influence the final output. Exploring alternative fusion strategies or incorporating additional validation steps could help address this limitation.

## Conclusions

In this experiment, we present an ensemble learning-based entropy-weighted average linear fusion method to address the limitations of individual segmentation models. We evaluate our approach on four diverse datasets and compare it with state-of-the-art methods.

For the Mask R-CNN model, which combines object localization and segmentation methods, we first use it to detect the varying structures of mitochondrial objects and then segment them into binary form. This algorithm’s results and limitations are shown in Figs 4 and 11. Likewise, We also employ a 3D U-Net model with data augmentation techniques to segment the mitochondrial regions of interest in the selected datasets. The results and limitations of this algorithm are shown in Figs 5 and 12. The Mask R-CNN model has several false negative cases, possibly due to its two-dimensional object localization approach focusing on individual objects rather than their 3D connectivity. Conversely, the 3D U-Net model has fewer false negatives but more false positives, emphasizing the connectivity of 3D objects rather than individual objects.

We also evaluated other advanced models, including Ddrnet-23, Segformer-B2, YoloV8, and YoloV9, but found that Mask R-CNN and 3D U-Net outperformed these alternatives on the four datasets. The Ddrnet-23 model, although effective at high-resolution segmentation, struggled with the fine-grained details in mitochondrial structures. Segformer-B2, which combines Transformer and CNN features, was less adept at capturing intricate mitochondrial details. YoloV8 and YoloV9, optimized for real-time object detection, were less effective in the pixel-wise segmentation required for our task.

To address the limitations of individual models, we propose an ensemble learning-based weight average fusion method. This approach combines the predicted outputs of both models by calculating weights using the entropy method. These weights are multiplied with their respective model predictions and summed to generate the final segmentation result, as shown in Figs 7 and 10. The visual results demonstrate the advantage of the fusion method over the individual deep neural network models. Our experiments demonstrate the state-of-the-art performance and robustness of our proposed methods, with higher scores in terms of *JI*_*F*_, *JI*_*B*_, *m*_*IoU*_, *DSC*, accuracy, precision, recall, AUROC, pixel accuracy, error rate, *TPR*, and *TNR* compared to other approaches.

In future work, we aim to explore integrating transformer-based deep neural network models to enhance mitochondria segmentation by capturing global dependencies and long-range interactions through self-attention mechanisms. Similarly, applying more sophisticated preprocessing techniques to handle noise and contrast variability, expanding the diversity of training datasets, and exploring more efficient and adaptive fusion strategies are potential directions.

## Supporting information

S1 File This file contains supplementary materials related to the study, including links to the datasets used. (PDF)
