# Supplementary material for: Deep learning-enhanced automated mitochondrial segmentation in FIB-SEM images using an entropy-weighted ensemble approach
Source: PLoS One. 2024 Nov 26;19(11):e0313000. doi: 10.1371/journal.pone.0313000 (PMC11593762; doi:10.1371/journal.pone.0313000)
Supplement: S1 File — (PDF) [file pone.0313000.s001.pdf]

Link of the datasets used in this study:

- https://www.epfl.ch/labs/cvlab/data/data-em/ (Lucchi (dataset-1))
- https://sites.google.com/view/connectomics (Lucchi++ (dataset-2))
- https://sites.google.com/view/connectomics (Kasthuri++ (dataset-3))
- https://github.com/MancaZerovnikMekuc/UroCell (UroCell (dataset-4))
